# Detailed characterisation of invasive aspergillosis in a murine model of X-linked chronic granulomatous disease shows new insights in infections caused by *Aspergillus fumigatus* versus *Aspergillus nidulans*

**DOI:** 10.3389/fcimb.2023.1241770

**Published:** 2023-09-01

**Authors:** Jill King, Ivy M. Dambuza, Delyth M. Reid, Raif Yuecel, Gordon D. Brown, Adilia Warris

**Affiliations:** ^1^ Medical Research Council (MRC) Centre for Medical Mycology, University of Exeter, Exeter, United Kingdom; ^2^ MRC Centre for Medical Mycology Aberdeen Fungal Group, Institute of Medical Sciences, University of Aberdeen, Aberdeen, United Kingdom; ^3^ Department of General Paediatrics, Royal Aberdeen Children’s Hospital, Aberdeen, United Kingdom; ^4^ Exeter Centre for Cytometrics, University of Exeter, Exeter, United Kingdom; ^5^ Iain Fraser Cytometry Centre, Institute of Medical Sciences, University of Aberdeen, Aberdeen, United Kingdom

**Keywords:** chronic granulomatous disease, *Aspergillus fumigatus*, *Aspergillus nidulans*, murine, infection, inflammation

## Abstract

**Introduction:**

Invasive aspergillosis (IA) is the most prevalent infectious complication in patients with chronic granulomatous disease (CGD). Yet, understanding of fungal pathogenesis in the CGD host remains limited, particularly with regards to *A. nidulans* infection.

**Methods:**

We have used a murine model of X-linked CGD to investigate how the pathogenesis of IA varies between *A. fumigatus* and *A. nidulans*, comparing infection in both X-linked CGD (gp91^-/-^) mice and their parent C57BL/6 (WT) mice. A 14-colour flow cytometry panel was used to assess the cell dynamics over the course of infection, with parallel assessment of pulmonary cytokine production and lung histology.

**Results:**

We observed a lack of association between pulmonary pathology and infection outcome in gp91^-/-^ mice, with no significant mortality in *A. nidulans* infected mice. An overwhelming and persistent neutrophil recruitment and IL-1 release in gp91^-/-^ mice following both *A. fumigatus* and *A. nidulans* infection was observed, with divergent macrophage, dendritic cell and eosinophil responses and distinct cytokine profiles between the two infections.

**Conclusion:**

We have provided an in-depth characterisation of the immune response to pulmonary aspergillosis in an X-linked CGD murine model. This provides the first description of distinct pulmonary inflammatory environments in *A. fumigatus* and *A. nidulans* infection in X-linked CGD and identifies several new avenues for further research.

## 1 Introduction

Invasive aspergillosis (IA) is the most prevalent infectious complication of patients suffering from chronic granulomatous disease (CGD) yet understanding of fungal pathogenesis in the CGD host remains limited, particularly with regards to *A. nidulans* infection (Marciano et al., 2015; King et al., 2016). CGD is a heterogeneous disease and *A. nidulans* appears to demonstrate a preference for the X-linked, rather than the autosomal recessive CGD host (Dotis and Roilides, 2011; Henriet et al., 2012; Henriet et al., 2013; King et al., 2016). However, the few published studies looking at *A. nidulans* infection have chosen to use a p47^-/-^ murine model of CGD (Chang et al., 1998; Bignell et al., 2005). This does not reflect the patient group at greatest risk of *A. nidulans* infection and may not accurately reflect disease pathogenesis in the X-linked CGD host. Differences in clinical presentation have been noted between *A. nidulans* and *A. fumigatus* infection in CGD patients (Segal et al., 1998; Henriet et al., 2012; King et al., 2016).

Our previous work has shown that *A. nidulans* lacks galactosaminogalactan (GAG), a cell wall polysaccharide that contributes to the virulence of *A. fumigatus* in immunocompromised hosts (Gravelat et al., 2013; Lee et al., 2015) and suppresses pro-inflammatory cytokine production in healthy (Gresnigt et al., 2014) and CGD cells *ex vivo (* Henriet et al., 2016). *In vitro* work has additionally demonstrated distinct differences in antifungal killing mechanisms between *A. nidulans* and *A. fumigatus (* Henriet et al., 2011) and in pro-inflammatory cytokine production (Henriet et al., 2012). However, it remains unclear to what extent these differences are reflected *in vivo* and how they relate to overall disease pathogenesis.

Using an appropriate disease model is important if we aim to understand why the X-linked CGD host is so exquisitely vulnerable to *A. nidulans* infection. The murine model of X-linked CGD developed by Pollock et al. through the targeted disruption of the gp91*^phox^* subunit of NADPH oxidase (referred to as gp91^-/-^) has been shown to closely mimic X-linked CGD both in terms of susceptibility to infection and inflammatory complications (Pollock et al., 1995; Morgenstern et al., 1997). We have used this model of X-linked CGD to investigate how the pathogenesis of IA varies between *A. fumigatus* and *A. nidulans*, comparing infection in both X-linked CGD (gp91^-/-^) mice and their parent C57BL/6 (WT) mice using fully molecularly characterised isolates of *Aspergillus* obtained from CGD patients. We employed a 14-colour flow cytometry panel adapted from that described by Misharin *et. al* (Misharin et al., 2013) to assess and compare the cell dynamics over the course of infection, with parallel assessment of pulmonary cytokine production and lung histology at set time points following infection.

## 2 Methods

### 2.1 *Aspergillus* isolates

Clinical isolates of *Aspergillus fumigatus* (V45-07) and *Aspergillus nidulans* (V44-46) from CGD patients with invasive aspergillosis, used in all *in vivo* experiments, were a kind gift from Prof Paul E. Verweij (Nijmegen, NL). *A. nidulans* strains overexpressing *Uge3* or *UgeB*, and the parent A26 strain, were kindly provided by Prof Don Sheppard and Dr Mark Lee, Montreal, Canada. Fluorescent *Aspergillus* Reporter (FLARE) conidia (Jhingran et al., 2012) were prepared from cultures of *A. fumigatus* Af293-dsRed conidia (a kind gift from Prof Tobias Hohl, Memorial Sloan-Kettering Cancer Centre, New York, USA).

All isolates were cultured in T-75 flasks (Grenier Bio-One) on glucose minimal media (GMM) agar slopes at 37°C and 5% CO_2_. For the auxotrophic *A. nidulans* galactosaminogalactan (GAG) mutants (An-Uge3 and An-UgeB), and their parent strain (A26), GMM was supplemented with 0.5 mg/ml biotin. Conidia were harvested after 7 to 10 days by washing flasks with phosphate-buffered saline (PBS, Gibco) supplemented with 0.05% Tween 80 (Fisher Scientific). The resulting conidial suspension was filtered through a 40 μm sterile cell strainer (Fisher Scientific), washed twice in PBS, counted and then diluted to the appropriate concentration in the relevant media.

*Aspergillus* hyphal layers were obtained by seeding 96-well filter plates (Merck Millipore) with 1 x 10 (Henriet et al., 2012) conidia of *A. fumigatus* (V45-07) or *A. nidulans* (V44-46) in 100μl RPMI and incubating plates at 37°CC and 5% CO_2_ for a minimum of 8 hours. The method for labelling Af293-dsRed conidia with the AF633 fluorophore was performed as described in Brunel et al. (2017).

### 2.2 Mice

All animals were bred and maintained at the Medical Research Facility, University of Aberdeen in accordance with the Home Office, UK, Animal Scientific Procedures Act, 1986. All experiments conformed to the terms and conditions of Home Office licences for research on animals and were approved by a local ethics review committee.

CYBB/C57BL/6 mice carrying a targeted disruption in the *Cybb* gene (Pollock et al., 1995) (referred to as gp91^-/-^) were kindly provided by Prof Steve Holland (National Institute of Allergy and Infectious Diseases, NIH, Bethesda, USA). These mice were backcrossed with our in-house C57BL/6J mice to minimise genetic variation between gp91^-/-^ mice and our wild-type (WT) C57BL/6J controls. Maintenance of genetic integrity was regularly checked by PCR.

### 2.3 Murine alveolar macrophages

Following humane sacrifice, alveolar macrophages were harvested from gp91^-/-^ and WT mice by broncho-alveolar lavage (BAL). Lavage was performed four times using 1 ml ice cold PBS supplemented with 5 mM ultrapure ethylenediaminetetraacetic acid (EDTA, Invitrogen Life Technologies) via an 18 gauge (G) intravenous cannula (Introcan-W, Braun) inserted into the trachea. Samples were centrifuged at 500 x g for 5 min at 4°C, supernatants discarded, and the resulting cell pellet re-suspended in 300 μl RPMI supplemented with 10% HI FBS. Cells from a minimum of six mice per group were pooled, counted, and their viability checked using Trypan Blue.

Cell suspensions were adjusted to between 1 x 10^5^ and 5 x 10^5^ cells/ml and 100 μl of the resulting cell suspension transferred to a 96-well filter plate (Merck Millipore). Plates were then incubated for three hours at 37°C and 5% CO_2_ to allow the macrophages to adhere. After three hours the media was refreshed before adding conidia to the wells at a ratio of 5 conidia to 1 AM. Plates were then incubated for 12 or 18 hours at 37°C and 5% CO_2_ before fungal viability was assessed using a colorimetric XTT assay as described by Henriet et al. (Henriet et al., 2011)

### 2.4 Murine bone marrow derived macrophages

Bone marrow derived macrophages (BMDMs) were obtained from gp91^-/-^ and WT mice using a protocol derived from that described by Zhang *et* al (Zhang et al., 2008) and Davies and Gordon (Davies and Gordon, 2005). After sacrifice by CO_2_ asphyxiation and gentle cervical dislocation, the skin was sterilised using 70% ethanol. The lower limb skin and soft tissue was then removed, and the tibia and femur placed on ice in RPMI. Bones were immersed in 70% ethanol for one minute before washing twice in ice cold sterile PBS. The epiphyses were removed using sterile scissors and the bone marrow canals flushed with 5 ml ice cold RPMI supplemented with 2 mM ultrapure EDTA (Invitrogen Life Technologies). The resulting bone marrow plugs were washed through a sterile 70 µM cell strainer (Fisher Scientific) and collected in a 50 ml polypropylene tube (Grenier Bio-One).

Cell suspensions were then centrifuged at 500 x g for ten minutes at room temperature, the supernatant discarded, and the resulting cell pellet resuspended in 50 ml of macrophage complete medium (Dulbecco’s Modified Eagle Medium (DMEM, Gibco) supplemented with 10% HI FBS, 1% Penicillin-Streptomycin (Sigma) and 20% L929-cell conditioned media. The resulting cell suspensions from each mouse were then divided between two 15 cm petri dishes (Corning™ Falcon™, Fisher Scientific) and incubated at 37°C with 5% CO_2_ for seven days to produce BMDMs. The media was refreshed every three days during this time. The method described by Mosser and Zhang (2008) was then used to generate classically activated macrophages from the differentiated BMDMs using 48-well plates.

For fungal killing assays, 1 x 10^6^ conidia in 250 μl DMEM plus 10% FBS were added to the appropriate wells of the 48-well plate (2 x 10^5^ BMDM/well; MOI 1:5) after a 24 or 46 hour LPS prime and the plates were then incubated for a further 18 hours at 37°C and 5% CO_2_. Following incubation, fungal viability was assessed using an XTT assay (Henriet et al., 2011).

### 2.5 Murine bone marrow derived neutrophils

Bone marrow derived neutrophils (BMDNs) were obtained from gp91^-/-^ and WT mice using the protocol described by Swamydas and Lionakis (2013). A cell count was performed on the resulting cell suspensions using a haemocytometer with Trypan Blue exclusion to check neutrophil viability prior to each experiment. Neutrophils suspensions were adjusted to the appropriate concentration for each experiment.

For neutrophil anti-hyphal assays, BMDNs were added to hyphal layers at a neutrophil to conidium ratio of 50 to 1 and the plates incubated for 12 hours at 37°C and 5% CO_2_. Following incubation, fungal viability was assessed using the XTT assay (Henriet et al., 2011).

FLARE conidia were added to 2 x 10^6^ BMDNs/ml in RPMI supplemented with 10% heat-inactivated FBS. Voriconazole was added at a final concentration of 2 μg/ml to inhibit conidial germination. After a 16-hour incubation at 37°C and 5% CO2, neutrophils and conidia were gently lifted from the wells using PBS with ultrapure EDTA, and the samples were prepared for flow cytometry analysis, following the procedures described in the flow cytometry section. The specific details of the antibodies and dyes used can be found in the supplementary data.

### 2.6 Experimental murine infections

Age-matched wild-type C57BL/6J and gp91-/- mice, between seven and thirteen weeks of age, were infected under anaesthesia by intra-tracheal instillation of 5 x 10^4^ *A. fumigatus* (V45-07) or *A. nidulans* (V44-46) conidia in 40 μl PBS. Following infection, mice were kept in sterile-isolator cages with autoclaved food and bedding, and acidified (gp91-/- mice) or sterile (WT mice) drinking water was provided *ad libitum*. Mice were weighed daily and monitored at least twice daily. Humane end-points were weight loss ≥ 30% or a clinical score ≥ 5 using our in house clinical monitoring system. Upon reaching the pre-determined end-points, mice were culled with a lethal overdose of pentobarbital sodium (Euthatal, Merial Animal Health, Ltd.) followed by gentle cervical dislocation.

Fungal burden was determined using lung homogenised in 1 ml sterile PBS. Serial 1 in 2 dilutions of a 100 μl volume of the resulting homogenate were plated on glucose minimal media and cultured at 37°C and 5% CO_2_. Colonies were counted daily for 5 days and colony forming units (cfu) per lung were calculated.

### 2.7 Histology

After sacrifice, the trachea was cannulated with an 18G (Introcan^®^-W, Braun) and 1 ml of a 1 to 1 volume per volume (v/v) solution of 2 M sucrose and optimal cutting temperature (OCT) compound (Tissue-Tek) was used to inflate the left lung. The lung was then excised, placed in a cryomold (ProSci Tech, Australia) containing OCT and then covered with OCT. Samples were immediately frozen at -20°C before transfer to an -80°C freezer on dry ice. Six to eight μm cryostat sections were obtained in triplicate from at least six different levels in each lung using a Leica CM1900 cryostat. Following sectioning, samples were allowed to dry overnight before dehydrating in 100% ethanol for 5 minutes ready for staining. Samples were either stained with haematoxylin and eosin (H&E) or a modified Grocott Methanamine Silver (GMS) stain (Accustain^®^ Silver Stain, Sigma-Aldrich) and finished with a haematoxylin counter stain. Slides were examined using a light microscope and selected slides uploaded to the Zeiss Axioscan Z1 slide scanner.

### 2.8 Cytokine assays

For cytokine analyses, homogenised lung in 1 ml PBS containing protease inhibitors (cOmplete™ ULTRA, mini, EDTA-free, Roche) was placed in 1.8 ml round bottomed Nunc CryoTubes (Thermo Scientific). Samples were then centrifuged at 3000 x g for 5 minutes at 4°C before removing the supernatant and freezing at -80°CC in 200 μl aliquots. Samples were analysed using multiplex magnetic bead assays from BioRad (custom 9-plex Bio-Plex Pro™ Assay) and R&D Systems (ELISA; Mouse IL-1α and IL-1β DuoSet; custom Luminex^®^ assay) as per manufacturers instructions.

### 2.9 Flow cytometry

For flow cytometry analysis, the left lung was minced into small pieces in a 15 ml polypropylene tube. The lung pieces were placed on ice in 5 ml RPMI. The samples were then transferred to a gentleMACS C-tube and incubated with Liberase TL and DNase I. The gentleMACS dissociator (Miltenyi Biotec GmbH) was used to process the samples according to the manufacturer’s instructions. Following dissociation, the lung digest samples were filtered through a 40 μm cell filter into a 50 ml polypropylene tube with ice-cold PBS used to wash through any residual sample. The samples were centrifuged at 300 x g for 5 minutes at 4°C, and the supernatant was discarded. Red blood cell lysis was performed by resuspending the cell pellet with BD Pharm Lyse (BD Biosciences Inc) for three minutes and stopped by adding ice-cold PBS. The resulting cell suspensions in FACS-Buffer (PBS with 2%BSA, 2mMEDTA) were counted and transferred to FACS tubes. Control samples were prepared by combining cell suspensions from each mouse, including a live-dead control. The cell suspensions were incubated with Fc receptor block and a fixable viability dye, followed by staining with a mixture of fluorochrome-conjugated antibodies (see **Supplementary Table 1**). Corresponding compensation (capture beads) and FMO controls were also created. All samples were analysed using the BD LSRFortessa (BD Bioscience Inc) flow cytometer equipped with 355m 405, 488, 561, and 640 nm Laser. Data analysis was performed by using FlowJo v10 software (BD Bioscience Inc). Cell populations were identified using a gating strategy adapted from Misharin et al. (2013), including CD3+ lymphocytes and NK cells (see **Supplementary Figure 1**). T-distributed stochastic neighbourhood embedding (t-SNE) analysis was performed using FlowJo v10.3 and the t-SNE plug-in The live, single-cell, CD45+ populations were selected, concatinated for each sample group (WT or gp91-/-), and down-sampled to 100,000 events.

### 2.10 Statistical analysis

GraphPad Prism for Mac OS X version 7.0c (GraphPad Software, La Jolla, California, USA) was used for statistical analysis and to display graphical results. Kaplan-Meier curves, analysed using Mantel-Cox log-rank analysis, were used to demonstrate survival. Comparisons between 2 groups were performed using a Student *t* test (normal distributed data) or the non-parametric Mann Whitney U test (non-Gaussian data). One-way ANOVA with Bonferroni post-test was used to compare >2 groups (normal distributed) or Kruskal-Wallis with Dunn’s multiple comparison (not normally distributed). p<0.05 was considered statistically significant. Error bars in graphical results represent standard error of the mean (SEM) unless otherwise stated.

## 3 Results

### 3.1 *A. nidulans* conidia demonstrate increased susceptibility to the antifungal activity of murine alveolar and bone marrow derived macrophages

**Figure 1:**
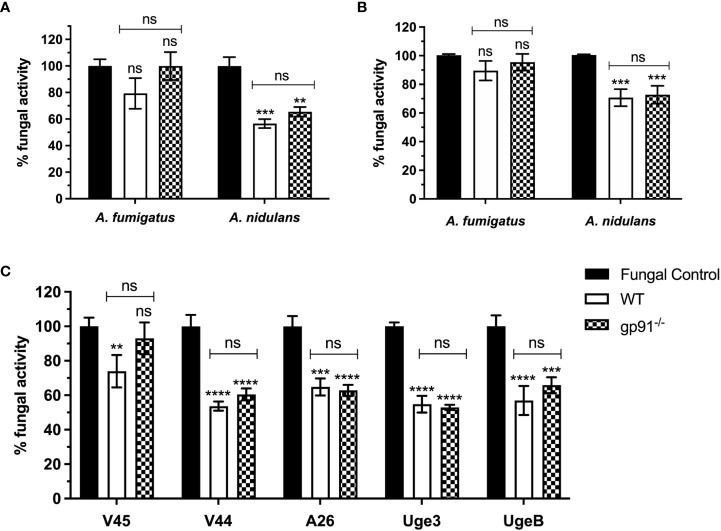
**(A)** Antifungal activity of murine alveolar macrophages (AMs) against *Aspergillus* conidia and **(C)** against *A. nidulans* strains overexpressing GAG. Fungal killing by WT and gp91^-/-^ AMs after 18 hours co-incubation with conidia, expressed as percentage fungal activity (XTT assay) compared to conidia only. Clinical isolates of *A. fumigatus* (V45) and *A. nidulans* (V44) were assessed alongside *A. nidulans* strains overexpressing *A. fumigatus Uge3* (Uge3) or *A. nidulans UgeB* (UgeB). A26 is the *A. nidulans* parent strain of the two modified GAG-producing *A. nidulans* strains. Results are shown from 3 independent experiments performed in triplicate using AMs obtained from 6 mice per group and a 1:5 cell to conidium ratio. **(B)** Antifungal activity of classically activated bone marrow derived macrophages (BMDMs) against *Aspergillus* conidia. Antifungal activity after 18 hours co-incubation with conidia, expressed as the percentage fungal activity (XTT assay) compared with conidia only. Results are shown from 3 independent experiments, in triplicate using BMDMs obtained from 3 mice per group and a 1:5 cell to conidium ratio. Statistical analysis was performed using 2-way ANOVA with Bonferroni post-test analysis. ns = non-significant, **p<0.01, ***p<0.001, ****p<0.0001.

Alveolar macrophages from gp91^-/-^ and WT mice demonstrated minimal antifungal activity against *A. fumigatus* following 18 hours co-incubation with resting conidia, with no significant difference between WT and gp91^-/-^ AMs (**Figure 1A**). In contrast, both gp91^-/-^ and WT AM significantly reduced the viability of *A. nidulans* conidia (p<0.01 for gp91^-/-^ AMs; p<0.001 for WT AMs) (**Figure 1A**). The anticonidial activity of activated BMDMs against *A. fumigatus* and *A. nidulans* conidia was comparable to our observations in AM, with both gp91^-/-^ and WT BMDM significantly reducing the viability of *A. nidulans* conidia (p<0.001; **Figure 1B**).

We assessed if this enhanced susceptibility of *A. nidulans* conidia to macrophage killing could be explained by the decreased galactosaminogalactan in the *A. nidulans* cell wall by using *A. nidulans* strains overexpressing *A. fumigatus Uge3* or *A. nidulans UgeB*. There was no significant difference observed (**Figure 1C**).

### 3.2 Antifungal activity of gp91^-/-^ BMDNs is maintained towards *A. fumigatus* hyphae but diminished against *A. nidulans* hyphae

**Figure 2:**
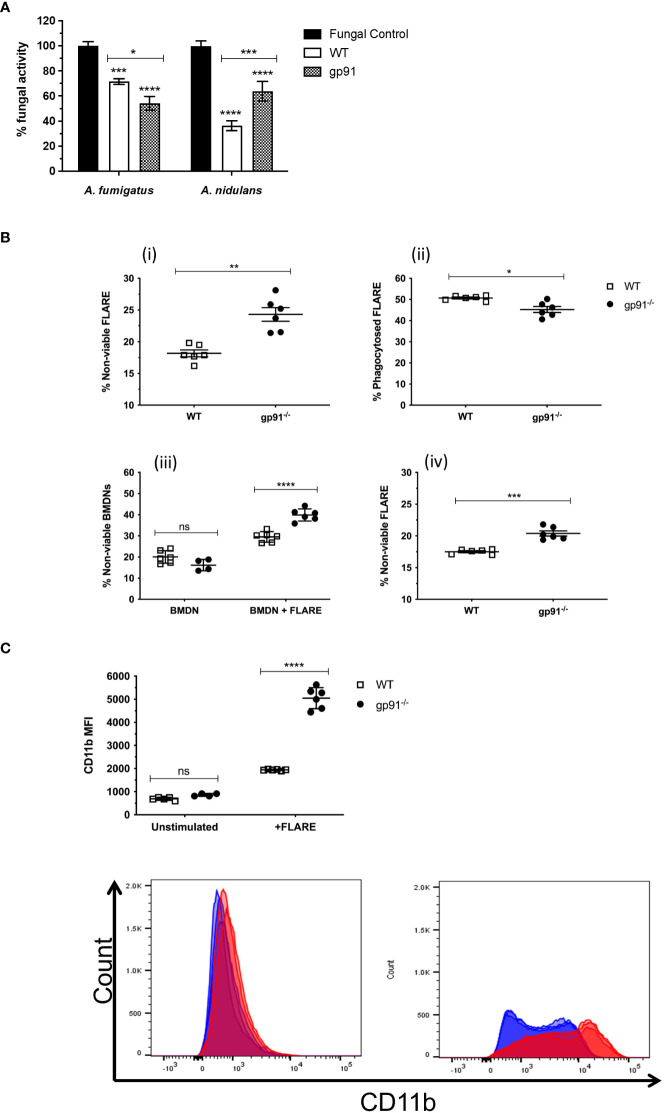
**(A)** Antifungal activity of bone marrow derived neutrophils (BMDNs) against *Aspergillus* hyphae. The antifungal activity of WT and gp91^-/-^ BMDNs against *A. fumigatus* and *A. nidulans* hyphae was assessed (XTT assay) after 12 hrs co-incubation and expressed as percentage fungal activity compared to hyphae alone. Data shown are from 3 independent experiments using BMDNs from 3 mice per group and a cell to conidium ratio of 50 to 1. Statistical analysis was performed using 2-way ANOVA with Bonferroni post-test analysis. ns = non-significant, *p<0.05, **p<0.01, ***p<0.001, ****p<0.0001. **(B)** Antifungal activity of BMDN against FLARE conidia. FLARE conidia were co-incubated with BMDNs from WT and gp91^-/-^ mice for 16 hours before staining and acquisition by flow cytometry. (i) Proportion of non-viable FLARE (AF633^+^, DsRed^-^) within WT and gp91^-/-^ neutrophils (CD11b^+^, Ly6G^+^), expressed as a percentage of total intracellular FLARE conidia. (ii) Percentage of intracellular FLARE conidia (AF633^+^, DsRed^+/-^, Ly6G^+^). (iii) Viability of either unstimulated WT and gp91^-/-^ neutrophils (BMDN), or following co-incubation with FLARE conidia (BMDN + FLARE), assessed using a fixable viability dye and expressed as a proportion of total neutrophils (CD11b^+^, Ly6G^+^). (iv) Non-viable FLARE (AF633^+^, DsRed^-^) expressed as a percentage of total FLARE (AF633^+^, DsRed^+/-^). Data shown from 2 independent experiments with 3 mice per group per experiment. Data in **(A, B, D)** were analysed using an unpaired t-test with Welch’s correction. Statistical significance in **(C)** was determined using two-way ANOVA with Bonferroni post-test analysis. ns = non-significant, *p<0.05, **p<0.01, ***p<0.001, ****p<0.0001. **(C)** Neutrophil CD11b MFI. CD11b MFI in WT and gp91-/- BMDNs before (unstimulated) or after (BMDN + FLARE) 16 hour co-incubation with FLARE conidia (BMDN + FLARE). Statistical significance determined using ANOVA with Bonferroni post-test analysis. ns = non-significant, ****p<0.0001.

Co-incubation of WT or gp91^-/-^ BMDNs with *Aspergillus* hyphae resulted in a significant reduction in fungal metabolic activity of *A. fumigatus* (p<0.001 and p<0.0001 respectively) and *A. nidulans* hyphae (p<0.0001 for both WT and gp91^-/-^). Surprisingly, co-incubation of gp91^-/-^ BMDN with *A. fumigatus* hyphae resulted in a greater reduction in metabolic activity than WT BMDN(p<0.05, **Figure 2A**). However, BMDNs from gp91^-/-^ mice were less effective against *A. nidulans* hyphae than WT BMDNs (p<0.001). The overall reduction in fungal activity by gp91^-/-^ BMDNs was similar between the two organisms however *A. nidulans* hyphae were significantly more susceptible to the antifungal activity of WT BMDNs than *A. fumigatus* (p<0.0001).

### 3.3 Preserved gp91^-/-^ anticonidial activity against *A. fumigatus* comes at the expense of increased neutrophil activation and death

To investigate the gp91^-/-^ BMDN anti-*Aspergillus* activity further, WT or gp91^-/-^ BMDNs were co-incubated with FLARE conidia. These *A. fumigatus* conidia emit a viability fluorophore (DsRed) that is extinguished upon conidial killing. The FLARE conidia are additionally coated in a second tracer fluorophore (AF633) to allow detection regardless of viability (Jhingran et al., 2012).

Surprisingly, gp91^-/-^ neutrophils were better able to kill *A. fumigatus* conidia than WT BMDNs, as demonstrated by the increased percentage of non-viable FLARE conidia after a 16-hour co-incubation with gp91^-/-^ neutrophils (p<0.01; **Figure 2B**). However, this came at the expense of increased neutrophil death, with gp91^-/-^ neutrophils significantly more likely to die following co-incubation with FLARE conidia than WT neutrophils (p<0.0001). This increased risk of cell death was not apparent in unstimulated gp91^-/-^ BMDNs.

CD11b is a cell surface marker that is up regulated following neutrophil activation (Kolaczkowska and Kubes, 2013). Both WT and gp91^-/-^ neutrophils demonstrated a significant shift in CD11b MFI following co-incubation with FLARE conidia (p<0.0001 for both WT and gp91^-/-^ compared with unstimulated BMDNs). However this increase was more marked in gp91^-/-^ BMDNs (p<0.0001; **Figure 2C**).

### 3.4 *A. fumigatus* infection in gp91-/- mice results in greater morbidity and mortality than *A. nidulans* infection

**Figure 3:**
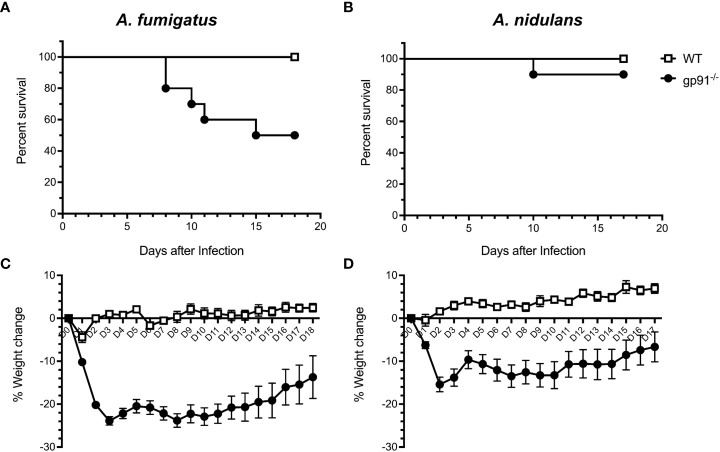
Natural course of *A. fumigatus* and *A. nidulans* infection in gp91-/- and WT mice. Kaplan-Meier survival of 8 to 11 week old WT (n = 10) and gp91-/- mice (n = 10) following intra-tracheal infection with 5x10^4^ conidia of **(A)** *A. fumigatus* or **(B)** *A. nidulans*. Statistical analysis performed using Mantel-Cox log-rank analysis, p=0.0114 and p=0.317 for *A. fumigatus* and *A. nidulans* survival curves respectively. Corresponding percentage change in weight following *A. fumigatus* and *A. nidulans* infection are demonstrated in **(C, D)**. Statistical analysis by unpaired student t-test show that gp91-/- mice suffer significantly more weight loss compared with WT mice, p<0.0001 in both infection models.

Significant early weight loss was seen following both *A. fumigatus* (p<0.0001) and *A. nidulans* (p<0.0001) infection in gp91^-/-^ mice compared with similarly infected WT mice, and was more pronounced and sustained in *A. fumigatus* infected gp91^-/-^ mice (**Figures 3C, D**). Mortality was 50% in *A. fumigatus* and 10% in *A. nidulans* infected gp91^-/-^ mice. None of the WT mice succumbed to infection (p=0.011 and p=0.317, resp., **Figures 3A, B**).

### 3.5 *A. fumigatus* and *A. nidulans* infection results in extensive inflammatory pathology and granuloma formation in the lungs of gp91^-/-^ mice

**Figure 4:**
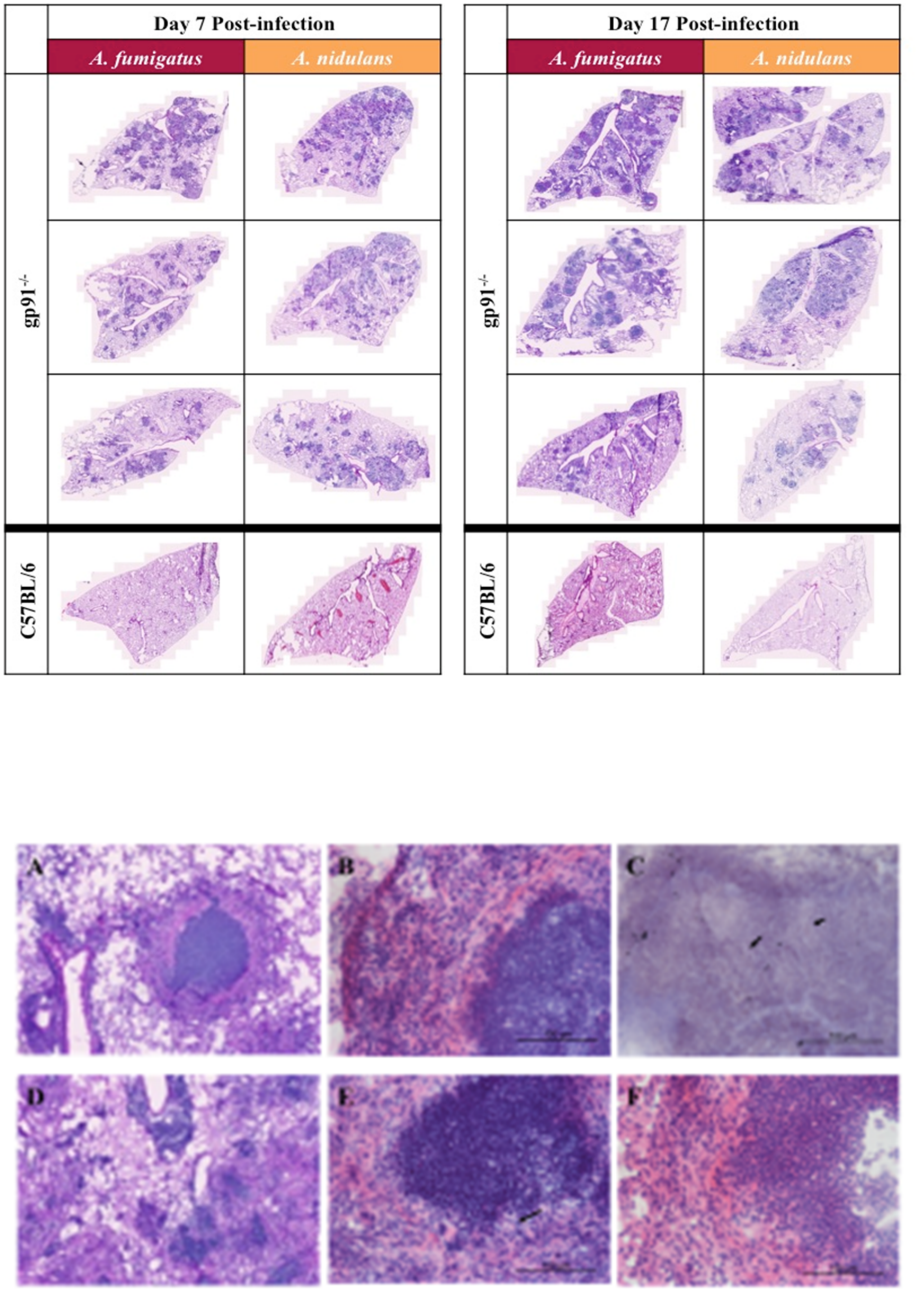
Pulmonary histology at 7d and 17d post-infection. Representative H&E stained sections from 3 mice per group showing extensive multi-focal pulmonary infiltrates at 7 and 17 days post-infection with *A. fumigatus* or *A. nidulans*. There was no evidence of pathology in sections from WT (C57BL/6) mice at any time point after infection, therefore only a single representative WT image is shown for each condition. Images were obtained using a Zeiss Axioscan Z1 slide scanner. Scale bars on each image represent 1000 µm. **(A–F)** Differences in granuloma formation between *A. fumigatus* and *A. nidulans* infection in gp91^-/-^ mice. Detail of an area of inflammation from a mouse infected with *A. fumigatus *A. and *A. nidulans* **(D)** at 17 days post-infection (H&E stained sections obtained using Zeiss Axioscan Z1 slide scanner, scale bars represents 200 μm). **(B)** Higher magnification view of granulomatous area from mouse infected with *A. fumigatus* at d17 post-infection showing a densely packed central accumulation of neutrophils, surrounded by an epitheliod cell layer and a more diffuse outer layer of lymphocytes, neutrophils and macrophages. **(E)** Shows a corresponding lesion from a mouse infected with *A. nidulans* at d17 post-infection with a similarly dense accumulation of neutrophils but lacking the outer cell layer organisation seen in *A. fumigatus* infection. **(F)** Demonstrates the epitheliod cell layer beginning to form around an area of neutrophil accumulation in a mouse infected with *A. nidulans* at d21 post-infection. **(B, E, F)** are H&E stained sections obtained using a Zeiss confocal light microscope, scale bars all represent 100 μm. Arrows indicate the sparse hyphal elements evident within granulomata on both H&E **(E)** and GMS **(C)** stained sections following *A. nidulans* and *A. fumigatus* infection respectively (scale bars represent 100 μm).

Fungal burden assessed by quantitative culture demonstrated low numbers of CFUs with a high degree of variability (between 0 and 600 CFU/lung) in both WT and gp91-/- mice up to d7 post-infection. Cultures on d17 were negative except for gp91-/- mice infected with *A. nidulans.* Histology samples showed no significant inflammatory change in the lungs of WT mice infected with either *A. fumigatus* or *A. nidulans* at any time point. In contrast, and despite the reduced morbidity and mortality seen in gp91^-/-^ mice infected with *A. nidulans*, pulmonary pathology was of comparable severity in both infections, with extensive pulmonary infiltrates at day 7 and evidence of early granuloma formation at 17 days in both *A. fumigatus* and *A. nidulans* infection (**Figure 4**).

There was some heterogeneity in pulmonary lesions within individual mice. However, in gp91^-/-^ mice infected with *A. fumigatus* lesions were typically well circumscribed, with a densely packed central neutrophil core, a surrounding layer of epitheliod cells, and an outer layer of more loosely organised macrophages, polymorphonuclear cells and lymphocytes (**Figures 4A–F**). The lesions present in mice infected with *A. nidulans* similarly had dense areas of neutrophil accumulation but these were less well delineated and a surrounding epitheliod cell layer was not evident until later in infection.

### 3.6 Excessive pulmonary neutrophil recruitment characterises both *A. fumigatus* and *A. nidulans* infection in gp91^-/-^ mice

**Figure 5:**
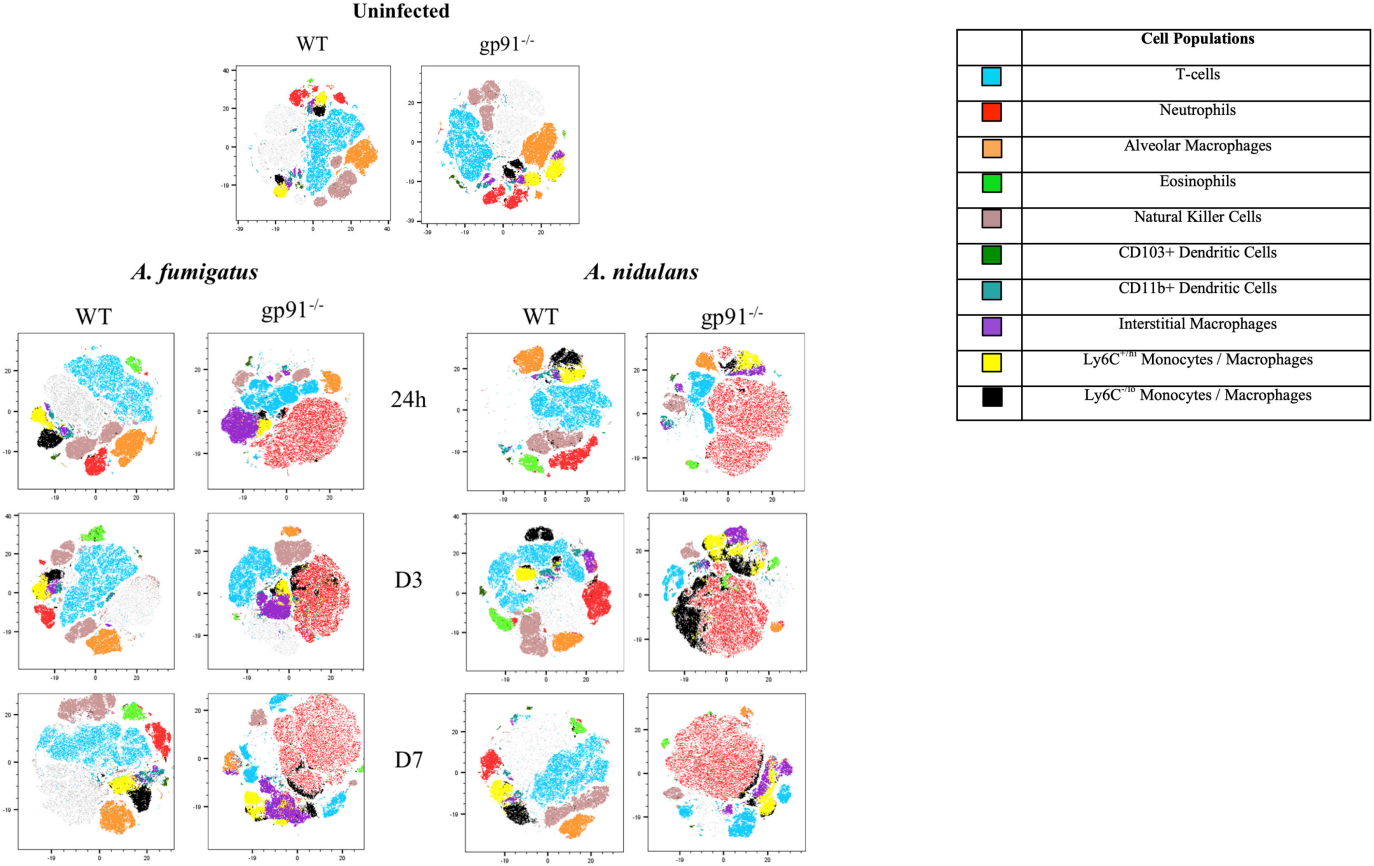
tSNE analysis of lung homogenates. tSNE analysis performed on all CD45+ cells after the exclusion of dead cells and doublets at each time point. Representative tSNE plots from a single uninfected WT and gp91-/- mouse are shown at the top of the page. For all subsequent time points samples from all 6 WT or gp91-/- mice in each experimental group were concatenated and down-sampled to 100,000 events prior to performing tSNE analysis. Cell populations identified using a traditional gating strategy (see materials and methods for gating strategy) were overlaid and are indicated in the legend.

The utilisation of t-distributed stochastic neighbourhood embedding (tSNE) to objectively analyse all CD45+ cell populations present in lung digest samples allows simultaneous visualisation of all analysed pulmonary cell populations in a two-dimensional map (**Figure 5**). Neutrophils clearly represent the predominant CD45+ cells present in the gp91^-/-^ lung at each time point following both *A. fumigatus* and *A. nidulans* infection. This is in contrast to WT mice where, similar to uninfected lung samples from both WT and gp91^-/-^ mice, T-cells remain the most prevalent cell type (**Figure 5**).

**Figure 6:**
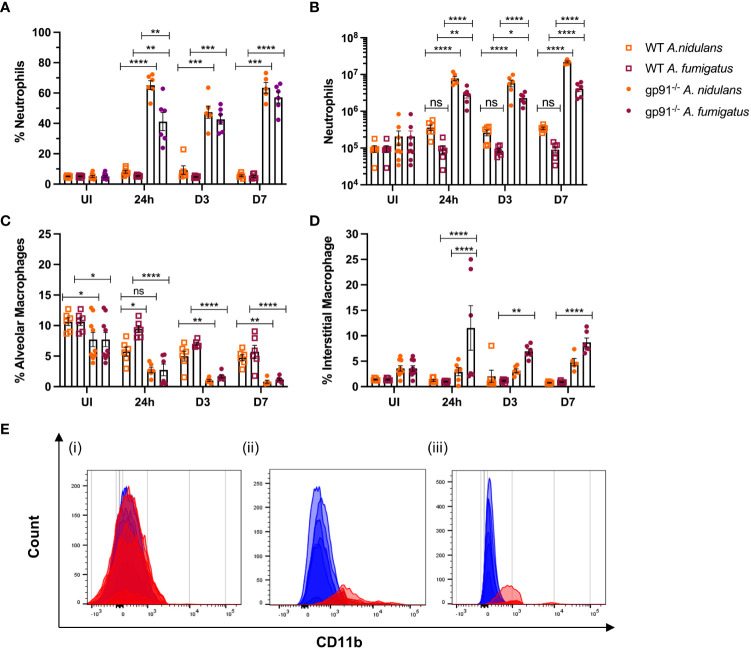
Pulmonary neutrophil recruitment and macrophage populations following *Aspergillus* infection in gp91^-/-^ and WT mice. **(A)** Neutrophils expressed as proportion of live CD45+ cells in uninfected (UI) WT and gp91^-/-^ mice and at 24 hrs, d3 and d7 after *A. fumigatus* and *A. nidulans* infection and **(B)** corresponding neutrophil counts at each time point **(C)** Proportion of live CD45+ cells identified as alveolar macrophages (CD45+, CD3-, CD11c+, Siglec F+) following *A. fumigatus* and *A. nidulans* infection. **(E)** Increasing alveolar macrophage CD11b MFI following *Aspergillus* infection. (i) Baseline CD11b expression in uninfected WT (blue) and gp91-/- (red) mice (MFI 236 ± 6 vs 269 ± 18 respectively, p = ns). (ii & iii) CD11b MFI in gp91-/- (red) mice compared with WT (blue) at 7d following (ii) *A. fumigatus* (MFI 2169 ± 216 versus 518 ± 28 respectively, p<0.0001) and (iii) *A. nidulans* infection (MFI 960 ± 50 versus 136 ± 6 respectively, p<0.0001). **(D)** Interstitial macrophage populations (CD45+, CD3-, CD11b+, MHCII+, CD64+, CD24-/lo) expressed as proportion of live CD45+ cells following infection with *A. fumigatus* or *A. nidulans*. Each time point represents an independent experiment, n = 6 per experimental group except uninfected gp91-/- samples where n = 9. Statistical analysis performed using ANOVA, ns = non-significant, *p<0.05, **p<0.01, ***p<0.001, ****p<0.0001.

Neutrophil numbers were significantly increased in gp91-/- lung digests within 24 hrs of infection, and at all subsequent time points in both infections compared with similarly infected WT mice and uninfected gp91-/- mice (**Figures 6A, B**). Neutrophil recruitment persisted over the 7 days following infection with no evidence of resolution in neutrophilic infiltration in gp91-/- mice. In contrast, there was no significant increase in neutrophil proportion or cell count in WT mice at any time following infection with either organism (**Figures 6A, B**; **Supplementary Figure 2**).

Neutrophil populations were phenotypically different in gp91-/- mice compared with WT mice. Similar to our findings during the *in vitro* experiments, neutrophil cell surface expression of the β2-integrin CD11b, was increased in uninfected gp91-/- mice compared with WT mice (16715 ± 436.6 versus 12271 ± 232.1 respectively, p<0.0001) and at all observed time points following both *A. fumigatus* and *A. nidulans* infection compared to similarly infected WT mice (p<0.0001 at 24 hours and 3 days following both *A. fumigatus* and *A. nidulans* infection; p<0.0001 at 7 days following *A. nidulans* infection and; p<0.05 at 7 days following *A. fumigatus* infection).

### 3.7 Loss of resident alveolar macrophages, and recruitment of pro-inflammatory macrophage populations, following *Aspergillus* infection in gp91-/- mice

The proportion of AM present in gp91^-/-^ lungs was significantly lower than similarly infected WT mice at each time point following both *A. fumigatus* and *A. nidulans* infection (**Figure 6C**; **Supplementary Figure 3**). Prior to infection, gp91^-/-^ mice had reduced AM populations compared with WT mice (p<0.05). However both WT and gp91^-/-^ mice had significant reductions in AM populations following *Aspergillus* infection when compared with corresponding healthy lung samples. This reduction persisted over the seven days following infection (p<0.0001 in gp91-/- mice at d7 following *A. fumigatus* and *A. nidulans* infection compared with uninfected samples) and is also clearly evident on tSNE analysis (**Figure 5**).

A clear phenotypic shift in CD11b expression in gp91-/- AM was observed following both infections, with these classically CD11b negative cells expressing increasing levels of the CD11b integrin (**Figure 6E**). This could not be accounted for by baseline differences in CD11b expression between uninfected WT and gp91-/- mice (CD11b MFI 236 ± 6 versus 269 ± 18 respectively, p = ns).

Alongside the reduction in resident AM populations following *A. fumigatus* and *A. nidulans* infection, pro-inflammatory macrophage populations were recruited to the gp91-/- lung. In *A. fumigatus* infected mice these infiltrating macrophages were predominantly interstitial macrophages (shown in purple on tSNE analysis, **Figure 5**), with subtle shifts in Ly6C^hi^ and Ly6C^lo^ monocyte/macrophage populations (shown in yellow and black respectively on tSNE plots, **Figure 5**). Within 24 hours of *A. fumigatus* infection in gp91-/- mice interstitial macrophage populations had increased significantly compared with uninfected gp91-/- mice and similarly infected WT mice (p<0.0001, **Figure 6D**). In contrast, following *A. nidulans* infection there was no significant change in the gp91-/- pulmonary interstitial macrophage population compared with uninfected gp91-/- mice and similarly infected WT mice (**Figure 6D**).

**Figure 7:**
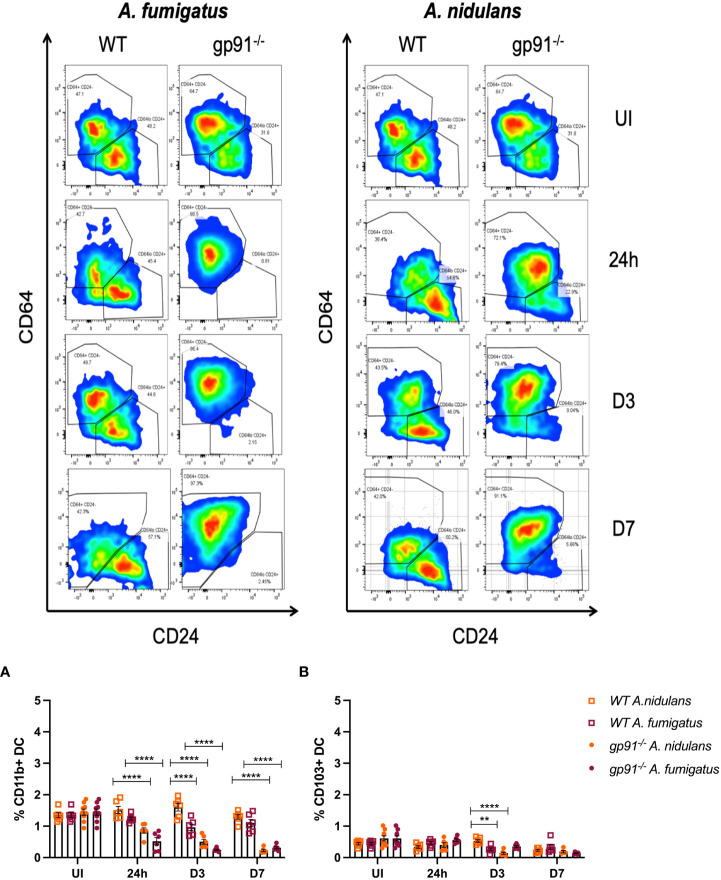
Representative FACS plots demonstrating increase in interstitial macrophages and loss of CD11b+ DC populations following *A. fumigatus* but not *A. nidulans* infection. Representative FACS plots from WT and gp91-/- mice at set time points following *A. fumigatus* (left panel) and *A. nidulans* (right panel) showing interstitial macrophage (CD45+, CD3-, CD11b+, MHCII+, CD64+, CD24-/lo, top-left quadrant of each plot) and CD11b+ dendritic cell (CD45+, CD3-, CD11b+, MHCII+, CD64-/lo, CD24+/lo, bottom-right quadrant) populations. An almost complete loss of CD11b+ DCs can be seen following *A. fumigatus* infection. **(A)** Pulmonary CD11b+ DCs and **(B)** Pulmonary CD103+ DCs following *A. fumigatus* and *A. nidulans* infection. Each time point represents an independent experiment with 6 mice per experimental group apart from uninfected (UI) gp91-/- samples that were obtained from two independent experiments (n = 9). Statistical analysis performed using ANOVA, **p<0.01, ****p<0.0001.

Following *A. fumigatus* infection the increase in interstitial macrophages was associated with almost complete loss of CD11b+ dendritic cells (DCs) in the gp91-/- lung (**Figures 5**, **6C–E**; p<0.0001 at all time points following infection compared with uninfected gp91-/- mice). In *A. nidulans* infection a significant reduction in CD11b+ DCs was observed in the gp91-/- lung (p<0.0001 at all time points post-infection compared with uninfected samples), however, a small population did remain evident on FACS analysis (**Figure 7**). Pulmonary CD103+ DCs were also reduced in gp91-/- mice at d3 and d7 following both *A. fumigatus* and *A. nidulans* infection (**Figures 7A, B**). DC populations did not change significantly in WT mice following either infection.

### 3.8 T-cell and natural killer cell populations are reduced in the gp91-/- lung following *Aspergillus* infection

**Figure 8:**
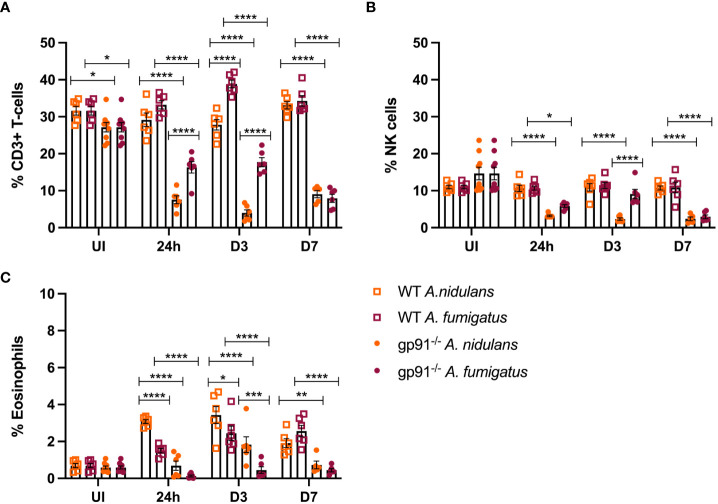
**(A)** Pulmonary T-cells following *Aspergillus* infection. T-cell populations (CD45+, CD3+) as proportion of live CD45+ cells following *A. fumigatus* and *A. nidulans* infection. **(B)** Pulmonary natural killer cell populations following *Aspergillus* infection. NK cell populations as proportion of CD45+ cells following *A. fumigatus* and *A. nidulans* infection in gp91-/- WT mice. **(C)** Pulmonary eosinophil populations following *Aspergillus* infection. Eosinophils (CD45+, CD3-, CD11c-, Siglec F+, CD24+, CD11b+, MHC II-) expressed as proportion of live CD45+ cells in WT (n = 6) and gp91-/- (n = 6) lungs following *A. fumigatus* and *A. nidulans* infection. Each time point represents an independent experiment with 6 mice per experimental group apart from uninfected (UI) gp91-/- samples that were obtained from two independent experiments (n = 9). Statistical analysis performed using ANOVA, ns = non-significant *p<0.05, **p<0.01, ***p<0.001, ****p<0.0001.

T-cells represented a significantly smaller proportion of CD45+ cells in the uninfected gp91-/- lung compared with uninfected WT mice (p<0.05, **Figure 8A**). Following infection with either *A*. *fumigatus* or *A. nidulans* the expansion in inflammatory macrophage and neutrophil populations resulted in significant proportionate reductions in T-cell populations in the gp91-/- lung compared with uninfected gp91-/- mice and similarly infected WT mice (p<0.0001 for all time points) whereas the proportion of CD3+ cells did not change in WT mice infected with *A. nidulans*. In contrast to the significant reduction in the CD3+ cell populations in gp91-/- mice following *A. fumigatus* infection, there was a significant expansion in CD3+ cells on d3 following *A. fumigatus* infection in WT mice compared with uninfected WT mice (p<0.001). Comparing *A. nidulans* and *A. fumigatus* infected gp91-/- mice, a comparatively greater proportion of CD3+ cells remained in the lungs of mice infected with *A. fumigatus* (**Figure 8A**). Natural killer (NK) cell populations were proportionately reduced in the gp91-/- lung following both *A. fumigatus* and *A. nidulans* infection. This was more evident following *A. nidulans* infection, however NK cell populations were significantly reduced compared to similarly infected WT and uninfected gp91-/- mice following both infections. There was no significant change in NK cell populations in WT mice following either infection (**Figure 8B**).

### 3.9 Wild-type mice mount an eosinophilic response to both *A. fumigatus* and *A. nidulans* infection

Wild-type mice developed eosinophilia, with significant increases in pulmonary eosinophils at 24 hours (p<0.05 and p<0.0001 for *A. fumigatus* and *A. nidulans* respectively), 3 days (p<0.0001 and p<0.0001), and 7 days (p<0.0001 and p<0.05) compared to uninfected WT mice. In both infections, maximum eosinophil response occurred on d3 and was resolving by d7 post-infection (**Figure 8C**). In contrast, there was no evidence of eosinophil recruitment in gp91-/- mice infected with *A. fumigatus* and the eosinophil population was almost completely absent on flow cytometry (**Figure 5**). Following *A. nidulans* infection, gp91-/- mice did demonstrate evidence of eosinophil recruitment at d3 post-infection compared with uninfected gp91-/- mice (p<0.001), however the response was significantly lower than that mounted by WT mice (p<0.0001, **Figure 8C**).

### 3.10 Pulmonary cytokine profiles reflect the highly pro-inflammatory environment in the gp91-/- lung

**Figure 9:**
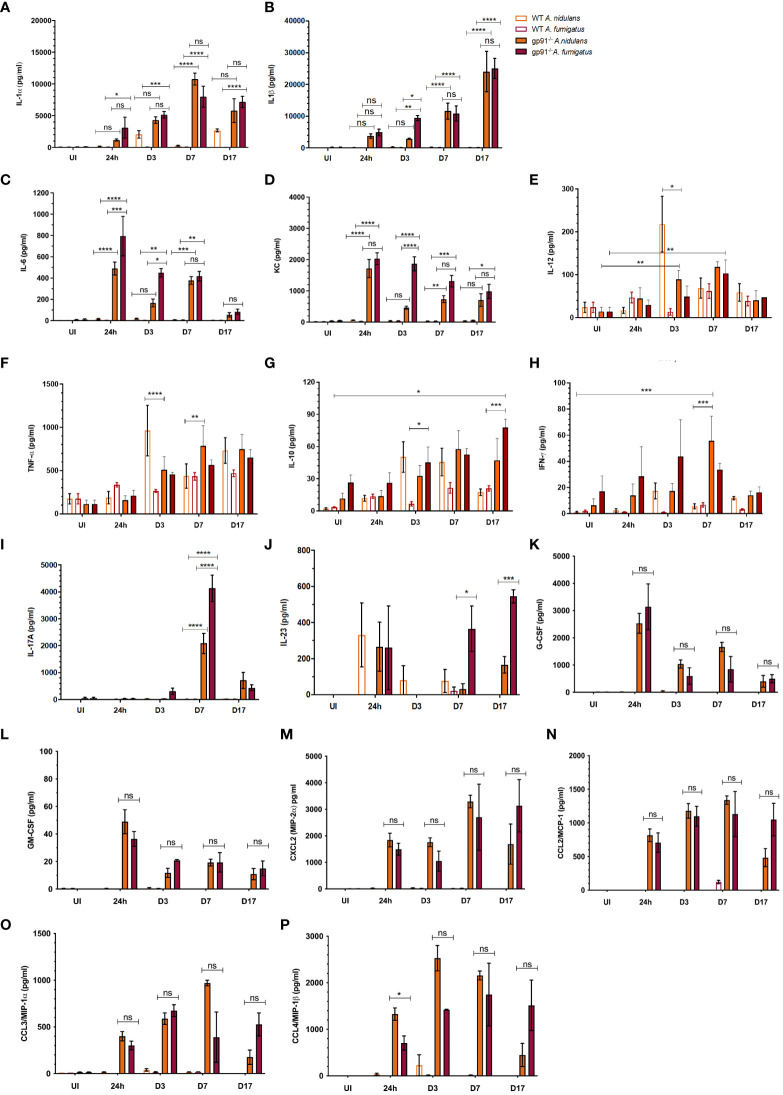
Pulmonary cytokine and chemokine responses following *Aspergillus* infection in WT and gp91-/- mice. **(A)** IL-1α determined using ELISA and multiplex magnetic bead assays; **(B)** IL-1β, **(C)** IL-6, **(D)** KC, **(E)** IL-12, **(F)** TNFα, **(G)** IL-10, **(H)** IFN-γ, and **(I)** IL-17 responses determined using multiplex magnetic bead assay. Each time point represents between one and three independent experiments with 6 mice per experimental group. Statistical analysis performed using ANOVA with Bonferroni post-tests, *p<0.05, **p<0.01, ***p<0.001, ****p<0.0001, ns = non-significant. **(J)** IL-23, **(K-M)** G-CSF, GM-CSF, CXCL2, and **(N-P)** CCL2 (MCP-1), CCL3 (MIP-1α), CCL4 (MIP-1β) assessed using multiplex magnetic bead assay. Between 2 and 12 samples were analysed for each condition per time point, representing between 1 and 2 independent experiments. Statistical significance determined using student t-test. ns = non-significant, *p<0.05, ***p<0.001.

A rapidly induced, and steadily increasing, IL-1 response was observed in gp91^-/-^ mice over the 17 days following infection with both *A. fumigatus* and *A. nidulans*. In gp91^-/-^ mice, pulmonary IL-1β in particular increased over 100-fold by day 17 following both *A. fumigatus* (25024 ± 3169 pg/ml, p<0.0001) and *A. nidulans* infection (24051 ± 6305 pg/ml, p<0.0001) compared with uninfected samples (150 ± 78 pg/ml) and showed no evidence of resolution (**Figure 9B**).

Pulmonary IL-1α increased steadily over the 7 days post-infections, increasing over 100-fold compared with uninfected mice (p<0.0001 for both infections), and peaked at d7 (**Figure 9A**). There was no significant difference in IL-1α or IL-1β response in gp91^-/-^ mice between *A. nidulans* and *A. fumigatus* infection, apart from on d3 following *A. nidulans* infection when IL-1β, but not IL-1α, was significantly lower (p<0.05) in mice infected with *A. nidulans* compared with *A. fumigatus*. However, by day 7 IL-1β concentrations resumed their parity with *A. fumigatus* infected gp91^-/-^ mice (**Figure 9B**).

IL-6 and KC levels peaked at 24 hrs and then gradually subsided, but remained elevated 17 days post-infection (**Figures 9C, D**). The initial magnitude of the KC response was similar between the two infections but subsequently dropped at d3 following *A. nidulans* infection, resulting in significantly lower KC (p<0.0001) compared with *A. fumigatus* infected mice at this time point. A drop in IL-6 was also evident at d3 post-infection in gp91-/- mice infected with *A. nidulans*, although the initial peak in IL-6 following *A. nidulans* infection was significantly lower than that seen in *A. fumigatus* infected mice (p<0.001). The gp91^-/-^ G-CSF and GM-CSF responses paralleled IL-6 and KC peaking at 24 hrs post-infection and subsequently resolving (**Figures 9K, L**). Whereas the CXCL2 response mirrored that of IL-1, increasing steadily over the first 7d post-infection (**Figure 9M**). No significant difference in G-CSF, GM-CSF or CXCL2 response was seen between *A. nidulans* and *A. fumigatus* infected gp91^-/-^ mice (**Figures 9K–M**). Significant increases were also seen in CCL2 (also known as monocyte chemoattractant protein (MCP)-1; p<0.0001 and p<0.01 for *A. nidulans* and *A. fumigatus* respectively), CCL3 (also known as macrophage inflammatory protein-1-alpha (MIP-1α); p<0.0001 for both infections) and CCL4 (also known as macrophage inflammatory protein-1-beta (MIP-1β); p<0.0001 for *A. nidulans* and p<0.01 for *A. fumigatus*) within 24 hrs of infection. The pattern of chemokine secretion was not significantly different between *A. fumigatus* and *A. nidulans* infection (**Figures 9N-P**). The IFN-γ response was minimal following both *A. fumigatus* and *A. nidulans* infection in gp91^-/-^ and WT mice. However, the increase in IFN-γ did reach significance at day 7 following *A. nidulans* infection in gp91^-/-^ mice compared with uninfected gp91^-/-^ mice (p<0.001) and was also significantly greater than similarly infected WT mice at this time point (p<0.001, **Figure 9H**).

### 3.11 Differential pulmonary IL-17 and IL-23 responses in gp91^-/-^ mice following *A. fumigatus* and *A. nidulans* infection may underpin their divergent pathology

Pulmonary IL-17 concentrations were significantly increased in gp91^-/-^ mice at d7 following both *A. fumigatus* (p<0.0001) and *A. nidulans* (p<0.0001) infection compared with uninfected gp91^-/-^ mice and similarly infected WT mice (p<0.0001 for both infections, **Figure 9I**). However, the IL-17 peak following *A. nidulans* infection was significantly lower than that following *A. fumigatus* infection (p<0.0001). *A. fumigatus* also induced significantly more of the Th-17 sustaining cytokine, IL-23, in gp91^-/-^ lung homogenates than *A. nidulans* (p<0.001, **Figure 9J**) suggesting *A. fumigatus* may preferentially induce a Th-17 promoting environment in the gp91^-/-^ lung.

### 3.12 Distinct cytokine responses induced by *A. nidulans* in wild-type mice

Wild-type mice showed a significant pulmonary IL-12 response at 3 days (p<0.01, **Figure 9E**) following *A. nidulans* infection. This response was significantly greater than that seen in similarly infected gp91^-/-^ lung homogenates (p<0.05). In addition, *A. nidulans* infection induced significantly more TNF-α than *A. fumigatus* at d3 and d7 post-infection (p<0.0001 and p<0.05 respectively) in WT mice. Similarly, in gp91-/- mice, *A. nidulans* induced significantly more TNF-α than *A. fumigatus* (p<0.01 at d3 and p<0.0001 at d7 post-infection). However, the peak in TNF-α in gp91-/- mice following *A. nidulans* infection appeared relatively delayed compared with WT mice, with maximum TNF-α seen at 7 days (784 ± 237 pg/ml) compared with a 3 days in WT mice (963 ± 293 pg/ml, **Figure 9F**). A gradual increase in IL-10 in gp91^-/-^ lung homogenates following *A. fumigatus* infection was observed reaching significance at d17 post-infection compared to WT mice (p<0.05). Both WT and gp91^-/-^ mice showed a small, gradual increase in pulmonary IL-10 following *A. nidulans* infection but this did not reach statistical significance (**Figure 9G**).

## 4 Discussion

This study outlines for the first time the pathogenesis of *A. nidulans* infection in an X-linked model of CGD, and additionally provides a direct comparison with *A. fumigatus* infection. It includes a detailed evaluation of the temporal dynamics in cell recruitment and cytokine production and, whilst it is clear that both *A. fumigatus* and *A. nidulans* are highly virulent in gp91^-/-^ mice, clear distinctions in pulmonary innate immune responses are evident between the two infections.

The most remarkable findings are; (1) the absence of significant mortality in *A. nidulans* infected gp91^-/-^ mice; (2) the lack of association between pulmonary pathology and infection outcome in gp91^-/-^ mice; (3) the overwhelming and persistent neutrophil recruitment and IL-1 release in gp91^-/-^ mice following both *A. fumigatus* and *A. nidulans* infection; (4) the divergent macrophage, dendritic cell and eosinophil responses between the two infections; (5) the distinct pulmonary cytokine profiles seen in *A. fumigatus* and *A. nidulans* infected gp91^-/-^ mice, particularly in relation to IL-17 and IL-23; (6) the reduced pulmonary T-cells in uninfected gp91^-/-^ mice; and (7) the unanticipated immune stimulatory effect of *A. nidulans* in WT mice resulting in increased pulmonary TNFα, IL-10 and IL-12.

The results from our *in vitro* work support the argument that ROS is dispensable for macrophage and neutrophil mediated host defence against *Aspergillus* conidia. In keeping with the results from previous studies (Morgenstern et al., 1997; Cornish et al., 2008; Henriet et al., 2011), we found murine gp91^-/-^ and WT macrophages demonstrate comparable antifungal activity against *A. fumigatus* and *A. nidulans* conidia. Mice with a conditional deletion of NOX2 in macrophages and/or neutrophils showed that *in vivo* NOX2 in alveolar macrophages has a non-redundant role to limit germination of *A. fumigatus* conidia and that for larger infective inocula neutrophil NOX 2 activity was key to clear the conidia. Although these results might seem to be in contrast with our results, the different genetic background is likely to explain the different observations (Idol et al., 2022). Our observations conflict with the study from Gresnigt et al. who found healthy human and murine macrophages were less able to kill *A. nidulans* conidia than *A. fumigatus (* Gresnigt et al., 2018). The authors attributed the impaired conidial killing to delayed phagocytosis of *A. nidulans* conidia and inhibition of LC3 recruitment to the phagolysosome by *A. nidulans (* Gresnigt et al., 2018). As LC3 recruitment to the phagosome is dependent on conidial swelling (Kyrmizi et al., 2013), and germination of *A. nidulans* conidia takes longer than *A. fumigatus* conidia [unpublished data], it is possible that the delayed LC3 recruitment demonstrated by Gresnigt et al. (Gresnigt et al., 2018) is the result of delayed conidial swelling rather than enhanced inhibition of LC3 recruitment by *A. nidulans*. Additionally, as LC3-associated phagocytosis (LAP) is reported to be impaired in CGD, the equivalent conidial killing by WT and gp91^-/-^ macrophages in our model suggests LAP is not the primary mechanism responsible for macrophage conidial killing of *Aspergillus* spp. We excluded that the reduced GAG in the *A. nidulans* cell wall is responsible for the enhanced killing. Further work is needed to better understand the mechanisms governing the enhanced susceptibility of *A. nidulans* to macrophage mediated conidial killing.

We demonstrated striking differences in the relative susceptibility of *A. nidulans* and *A. fumigatus* to innate host defence mechanisms, with *A. nidulans* conidia and hyphae exhibiting enhanced susceptibility to the healthy host phagocytes. gp91^-/-^ neutrophils were less able to inflict damage on a preformed *A. nidulans* hyphal layer than WT neutrophils, suggesting defective anti-hyphal host defence in CGD underpins the pathogenicity of *A. nidulans* in the CGD host. In contrast with other studies (Rex et al., 1991; Ahlin et al., 1997; Henriet et al., 2011; Gazendam et al., 2016), we found that gp91^-/-^ neutrophils maintained their antifungal efficacy against *A. fumigatus*. All these studies used PMNs from CGD patients and it is possible there are differences in hyphal killing between murine and human neutrophils. However, a recently published study by Lee et al. found that, in keeping with our murine model, neutrophils from a CGD patient were equally capable of restricting *A. fumigatus* hyphae as healthy controls but were less able to inflict damage on *A. nidulans* hyphae (Lee et al., 2015). In their study, impaired NETosis in CGD resulted in impaired antifungal host defence as *A. nidulans* hyphae demonstrated enhanced susceptibility to the neutrophil lysates formed during NETosis, with *A. fumigatus* hyphae being relatively resistant to these due to the presence of GAG on the hyphal cell wall (Lee et al., 2015).

*A. nidulans* infection in gp91^-/-^ mice resulted in significantly less weight loss and lower mortality than *A. fumigatus* infection. However, this belied the extensive pulmonary pathology and significant inflammatory response induced by *A. nidulans* infection in the gp91^-/-^ mice. The extensive pulmonary pathology despite apparent clinical recovery in our murine model supports the indolent clinical presentation of *A. nidulans* infection in CGD patients (Segal et al., 1998; Henriet et al., 2012; Henriet et al., 2013). Previous studies of IA in CGD have clearly described extensive pulmonary inflammation and granuloma formation (Morgenstern et al., 1997; Bignell et al., 2005; Cornish et al., 2008) and our histopathological assessment is in agreement with this. Using slide scanner technology to view whole lung sections showed that the lesions were distributed throughout the lung, and allowed an appreciation of the heterogeneity of pulmonary lesions following *A. fumigatus* and *A. nidulans* infection. The observed discrepancies in granuloma formation between *A. nidulans* and *A. fumigatus* infection are intriguing and might be associated with variation in disease outcome as has been described in *M. tuberculosis* infection (Irwin et al., 2015).

Using a 14-colour flow cytometry panel allowed us to simultaneous assess the dynamics of innate immune cell recruitment following pulmonary challenge with *Aspergillus* in a murine X-linked CGD model alongside the concomitant determination of key cytokine and chemokine responses, providing a global picture of the innate host response to pulmonary *Aspergillus* infection in X-linked CGD. An exaggerated and persistent, predominantly neutrophilic, pulmonary cellular infiltrate in gp91^-/-^ mice following both *A. fumigatus* and *A. nidulans* infection was observed. Neutrophil recruitment is a tightly controlled, multi-step process and requires the co-ordinated induction of a variety of cytokines and chemokines. G-CSF and the CXC chemokine receptor 2 (CXCR2) ligands, KC and MIP-2α, were significantly induced during both infections. Whilst CXCR2 mediated signalling is essential for effective antifungal host defence (Mehrad et al., 1999; Mehrad et al., 2002; Bonnett et al., 2006) the exaggerated responses seen in gp91^-/-^ mice likely contribute to excessive neutrophil recruitment and host damage. Interestingly, whilst KC and G-CSF responses gradually resolved over the 17 days following infection, MIP-2α remained elevated, suggesting MIP-2α may play a role in the sustained neutrophil recruitment seen at later time points in our model.

Exaggerated IL-1 responses have previously been demonstrated in X-CGD myeloid cells in response to a variety of agonists (Bagaitkar et al., 2015), and stimulation with *A. fumigatus* and *A. nidulans* have shown excessive IL-1β responses both *in vitro* (Gresnigt et al., 2014) and *in vivo* (Morgenstern et al., 1997). However, in contrast to the seminal description of IA in CGD mice by Morgenstern et al (Morgenstern et al., 1997) where IL-1β lung mRNA on Northern blot analysis started to decrease one week after *A. fumigatus* infection, we found no evidence of resolution in pulmonary IL-1β at 7 or 17 days following *A. fumigatus* and *A. nidulans* infection. The IL-1α responses did, however, peak at 7 days post-infection. Whilst IL-1α, rather than IL-1β, has been shown to be critical for early neutrophil recruitment in both *Aspergillus* infection (Bagaitkar et al., 2015; Caffrey et al., 2015) and sterile peritoneal inflammation (Bagaitkar et al., 2015), Bagaitkar et al. found neutralisation of IL-1β significantly reduced neutrophil recruitment at 72 hours after stimulation, suggesting IL-1β may play a role in modulating neutrophil recruitment at later time points (Bagaitkar et al., 2015). Taken together these findings support the hypothesis that gp91^-/-^ mice are unable to terminate IL-1 mediated inflammation, and that interventions to modify the IL-1 response following *Aspergillus* infection may be beneficial to the CGD host. In contrast to previously published *in vitro* work (Henriet et al., 2016), we did not detect a significant difference in IL-1β or IL-1α between *A. nidulans* and *A. fumigatus* infection. Galactosaminogalactan (GAG), a cell wall polysaccharide present in *A. fumigatus* but not *A. nidulans*, has been shown to have potent anti-inflammatory effects in murine models of IA, reducing neutrophil recruitment (Fontaine et al., 2011), concealing β-glucans from the host immune system (Gravelat et al., 2013), and reducing IL-1R1 signalling *via* the induction of IL-1 receptor antagonist (IL-1Ra) (Gresnigt et al., 2014; Henriet et al., 2016). However the role of GAG in the hypoxic lung environment following *in vivo* infection might be minimal as hypoxia interferes with GAG production in *A. fumigatus (* Shepardson et al., 2013). This could explain the lack of differential IL-1 production seen in our model.

The increased neutrophil expression of the β_2_-integrin, CD11b in gp91^-/-^ mice, both before and after *Aspergillus* infection, is in keeping with our *in vitro* findings and is interesting from several perspectives. Increased cell surface expression of CD11b is a hallmark of activated neutrophils (Kolaczkowska and Kubes, 2013) and, although not previously reported, increased neutrophil activation during IA in CGD is not surprising. CD11b is critical component of complement receptor 3 (CR3) and has been shown to play an important role in neutrophil recognition of β-glucan in *A. fumigatus* (Gazendam et al., 2016). CR3-mediated phagocytosis has been shown to induce neutrophil apoptosis in an NADPH oxidase dependent manner and has been found to be defective in CGD and DPI-treated neutrophils (Coxon et al., 1996). Although neutrophil recruitment is essential for effective antifungal host defence, excessive and sustained neutrophil recruitment contributes to tissue damage and inflammatory pathology (Medzhitov, 2008; Jones et al., 2016). Neutrophil apoptosis is one mechanism of limiting this inflammation and appears defective in CGD, (Coxon et al., 1996; Brown et al., 2003; Frasch et al., 2008; Fernandez-Boyanapalli et al., 2010). Additionally, suppression of β_2_-integrin mediated signalling via sialic acid binding Ig-like lectin E (Siglec-E) has been shown to negatively regulate pulmonary neutrophil recruitment in an NADPH oxidase dependent manner (McMillan et al., 2013; McMillan et al., 2014) suggesting mechanisms to limit neutrophil recruitment are impaired in CGD. How the increased CD11b expression seen in gp91^-/-^ neutrophils impacts on IA disease pathogenesis remains unclear. However, the identification of these activated neutrophils in the lung, together with the multiple deficiencies that have been identified in CD11b-mediated signalling in CGD, suggest inappropriately sustained neutrophil recruitment and activation, and failure to effectively terminate neutrophilic inflammation.

Alveolar macrophages were significantly reduced in gp91^-/-^ mice following both *A. fumigatus* and *A. nidulans* infection. As AM act to suppress DC and T-cell activation, preventing inappropriate inflammation (Thepen et al., 1989; Holt et al., 1993; Blumenthal et al., 2001), the reduction of AM in the gp91^-/-^ mice is likely to contribute to excessive inflammation, as also reflected in the recruitment of inflammatory macrophages populations.

We found no significant difference in IL-1α or CXCL1 expression between *A. fumigatus* and *A. nidulans* infection, despite the relative lack of Ly6C^hi^ monocyte recruitment in *A. fumigatus* infected gp91^-/-^ mice. The relatively increased TNF-α seen in gp91^-/-^ mice following *A. nidulans* compared with *A. fumigatus* infection may play a role in the enhanced recruitment of Ly6C^hi^ monocytes (Park et al., 2010). The substantial reduction of Ly6C^hi^ monocytes in *A. fumigatus* infected gp91^-/-^ mice as demonstrated in our model, is therefore likely to significantly impair host defence (Phadke et al., 2007; Park et al., 2010). The increased Ly6C^lo^ monocyte/macrophage population observed in *A. nidulans* infected gp91^-/-^ mice at day 3 post-infection correlates with a period of reduced cytokine expression and apparent clinical recovery and may contribute to the divergence in host responses between *A. fumigatus* and *A. nidulans* infected mice at this time point.

The finding of a reduced baseline CD3+ cell population in murine gp91^-/-^ lungs was unexpected. In the study by Cruz et al. (Cruz et al., 2013), looking at T-cell responses to *A. fumigatus* infection in gp91^-/-^ mice, the authors reported baseline T-cell populations in gp91^-/-^ spleens were similar to wild-type (C57BL/6) mice. Reductions in specific T-cell populations have however been documented with reduced CD8+ T cells in gp91^-/-^ spleens noted in the model by Cruz et al. and a reduction in αβ T-cells in uninfected p47^-/-^ murine lungs described by Romani et al. (Romani et al., 2008; Obar et al., 2016). The discovery of phagocyte-type NADPH oxidase expression in T-cells (Jackson et al., 2004), and a reported Th1/Th17 bias in CGD T-cells stimulated *ex vivo* (Jackson et al., 2004; Horváth et al., 2011; Cachat et al., 2015), suggest dysfunction in the adaptive immune response in CGD may play an additional role in driving inflammatory pathology. The proportionate reductions in T-cell populations following *Aspergillus* infection in gp91^-/-^ mice are partially an artefact due to the hugely expanded neutrophil populations but the relative suppression of T-cells in *A. nidulans* compared with *A. fumigatus* infection is interesting and suggests divergence in the adaptive responses.

The distinct cytokine profiles observed in the two infections paint a picture of two divergent innate lymphoid cell (ILC) phenotypes. A broadly type 3 response with significantly more IL-17, IL-23 and IL-6 in *A. fumigatus* infected gp91^-/-^ lungs, and a comparatively increased IL-12, IFN-γ and TNF-α expression in *A. nidulans* infected mice compatible with a type 1 response. While a type 1 ILC response is regarded as protective during *Aspergillus* infection (Chai et al., 2010; van de Veerdonk and Netea, 2010; ROmani, 2011), the relative contribution of Th-17 immunity (type 3 ILC response) remains controversial (Zelante et al., 2007; ROmani et al., 2008; Werner et al., 2009; Chai et al., 2010; Jolink et al., 2017). The differences in innate immune cytokine environments may explain the subsequent differences in granuloma formation and mortality between the two infections. The complex interplay between innate and adaptive immune responses may be additionally compounded by the ability of *Aspergillus* to direct the adaptive host response to its own advantage. Rivera et al. found *A. fumigatus* was able to subvert T-helper responses in a dectin-1 dependent manner by reducing the expression of IFN-γ and IL-12, suppressing Th-1 responses and promoting Th-17 differentiation (Rivera et al., 2011). If this response is specific to *A. fumigatus* it could help to explain the divergent cytokine responses between our two infection models.

In summary, we have provided an in-depth characterisation of the immune response to pulmonary aspergillosis in an X-linked CGD murine model, with direct comparison between infection caused by *A. fumigatus* and *A. nidulans*, in order to better understand the pathogenesis of IA in the CGD host (see **Supplementary Table 2**). Utilising a novel 14-colour flow cytometry technique has allowed a comprehensive characterisation of immune cell recruitment following infection and we have additionally correlated this with host cytokine responses and pulmonary pathology. It is evident that both *A. fumigatus* and *A. nidulans* infection result in significant disease, with extensive pulmonary pathology and a marked inflammatory response. However, there are distinct differences in pulmonary macrophage, dendritic cell and eosinophil recruitment between the two infections, and divergent inflammatory environments; with *A. fumigatus* infection typified by a predominantly type 3 response characterised by high levels of IL-6, IL-17 and IL-23 and early granuloma formation but high mortality; and *A. nidulans* infection demonstrating a mixed inflammatory response with elements of type 1, type 2 and type 3 responses, delayed granuloma formation and reduced mortality despite significant pathology. This provides the first, intriguing description of distinct pulmonary inflammatory environments in *A. fumigatus* and *A. nidulans* infection in X-linked CGD and identifies several new avenues for further research.

## Data availability statement

The original contributions presented in the study are included in the article/**Supplementary Material**. Further inquiries can be directed to the corresponding author.

## Ethics statement

All experiments conformed to the terms and conditions of Home Office licences for research on animals and were approved by a local ethics review committee. The study was conducted in accordance with the local legislation and institutional requirements.

## Author contributions

JK, ID, DR, and RY performed the work. JK wrote a first draft of the manuscript. AW and GB conceptualised the work. AW drafted the final version of the manuscript. All authors contributed to the article and approved the submitted version.
