# Supplementary material for: Detailed characterisation of invasive aspergillosis in a murine model of X-linked chronic granulomatous disease shows new insights in infections caused by Aspergillus fumigatus versus Aspergillus nidulans
Source: Front Cell Infect Microbiol. 2023 Sep 1;13:1241770. doi: 10.3389/fcimb.2023.1241770 (PMC10505440; doi:10.3389/fcimb.2023.1241770)
Supplement: Supplementary file 1 [file DataSheet_1.docx]

**Suppl. Table 1. Antibodies used in the 14-colour flow cytometry panel.**

| **Antigen** | **Clone** | **Fluorochrome** | **Manufacturer** |
| --- | --- | --- | --- |
| CD45 | 30-F11 | BUV395 | BD Biosciences |
| CD3 | 17A2 | PE-Vio770  APC-Cy7 | Miltenyi Biotec  BD Biosciences |
| NK1.1 | PK136 | PerCp-Cy5.5 | BD Biosciences |
| CD11b | M1/70 | BUV737 | BD Biosciences |
| CD11c | HL3 | BV711 | BD Biosciences |
| CD24 | M1/69 | BV605 | BD Biosciences |
| CD64a/b | X54-5/7.1 | PE | BD Biosciences |
| CD103 | M290 | BV510 | BD Biosciences |
| Ly6G | 1-A8 | FITC | BD Biosciences |
| Ly6C | AL-21 | APC | BD Biosciences |
| Siglec F | E50-2440 | PE-CF594 | BD Biosciences |
| F4/80 | T45-2342 | BV421 | BD Biosciences |
| MHC II (I-A/I-E) | M5/114.15.2 | BV650 | BD Biosciences |
| Fixable viability dye |  | eFluor780 | eBioscience |

Suppl. Figure 1. 14-colour flow cytometry gating strategy. Cells were isolated from enzymatically digested mouse lungs and, after the exclusion of dead cells, doublets and debris, immune cells were identified by CD45 staining (A → D). A sequential gating strategy based on that described by Misharin et al.273 was then used to identify; (E) T-cells (CD45+, CD3+); (F) Neutrophils (CD45+, CD3-, CD11b+, Ly6G+); (I) Alveolar macrophages (CD45+, CD3-, CD11c+, Siglec F+) and Eosinophils (CD45+, CD3-, CD11c-, Siglec F+); (M) CD103+ dendritic cells (CD45+, CD3-, Siglec F-, CD11c+, CD103+); (P) NK cells (CD45+, CD3-, Siglec F-, CD11bint, NK1.1+); and from the remaining NK1.1 negative population the remaining CD11b+ populations were assessed for MHC II, CD64, Ly6C and CD24 expression to identify; (R) Ly6Chi monocytes and macrophages (CD11b+, MHC II-, CD64+/-, Ly6Chi) and Ly6Clo monocytes and macrophages (CD11b+, MHC II-, CD64-, Ly6Clo) and; (S) Interstitial macrophages (CD11b+, MHC II+, CD64+/lo, CD24-/lo) and CD11b+ dendritic cells (CD11b+, MHC II+, CD64-/lo, CD24+). Additional analysis of neutrophils, alveolar macrophages, eosinophils and CD103+ dendritic cells were performed as detailed in G, H, J, K, L, N and O to confirm they represented uniform populations and to delineate additional attributes of interest such as CD11b expression. FSC, forward scatter; SSC, side scatter; A, area; H, height; W, width.


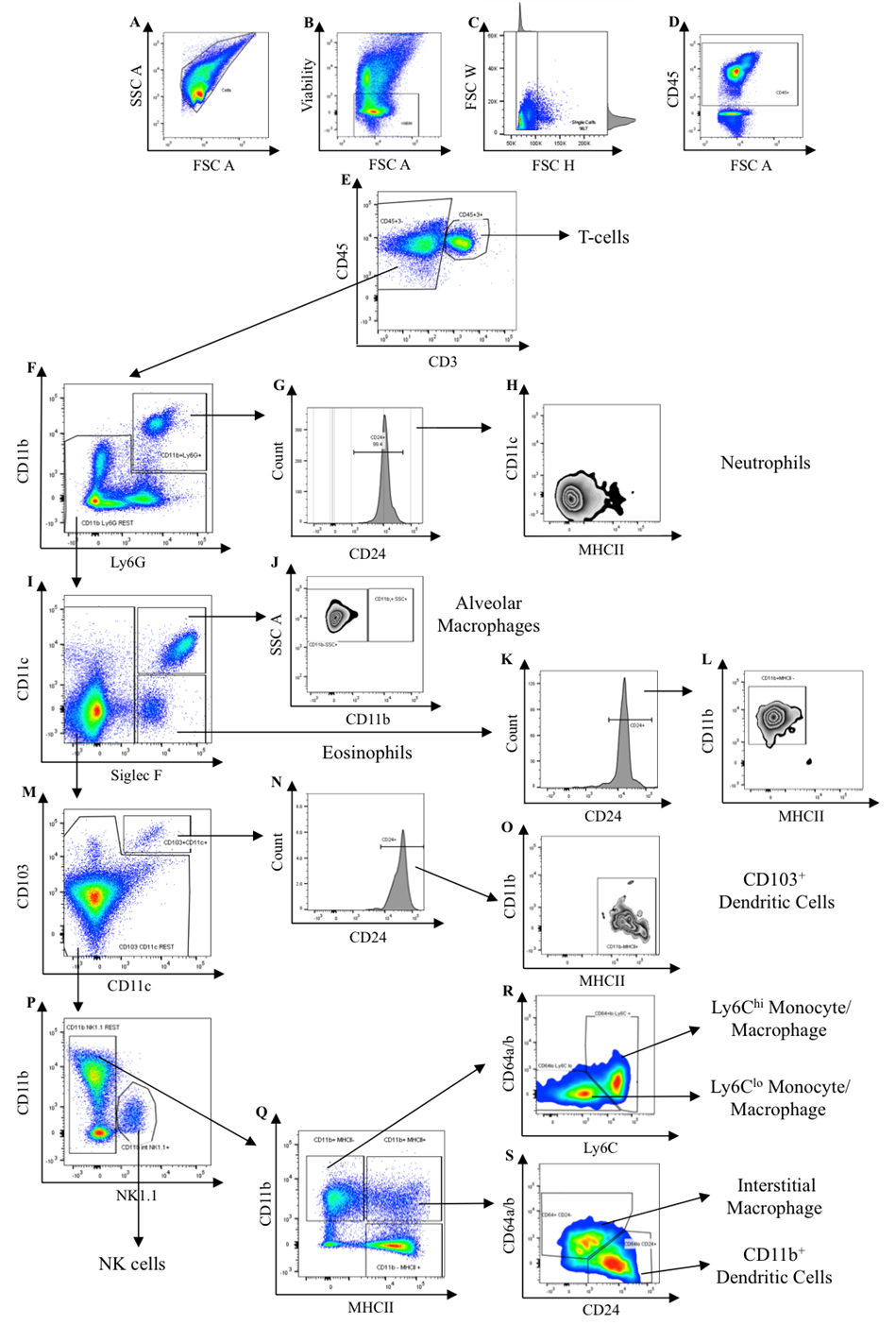


Suppl. Figure 2. Neutrophil recruitment following *Aspergillus* infection. Representative FACS plots from WT and gp91­^-/-^ mice at each time point following *A. fumigatus* (left panel) or *A. nidulans* infection (right panel). Neutrophils gated as CD45+, CD3-, CD11b+, Ly6G+ after excluding dead cells and doublets.


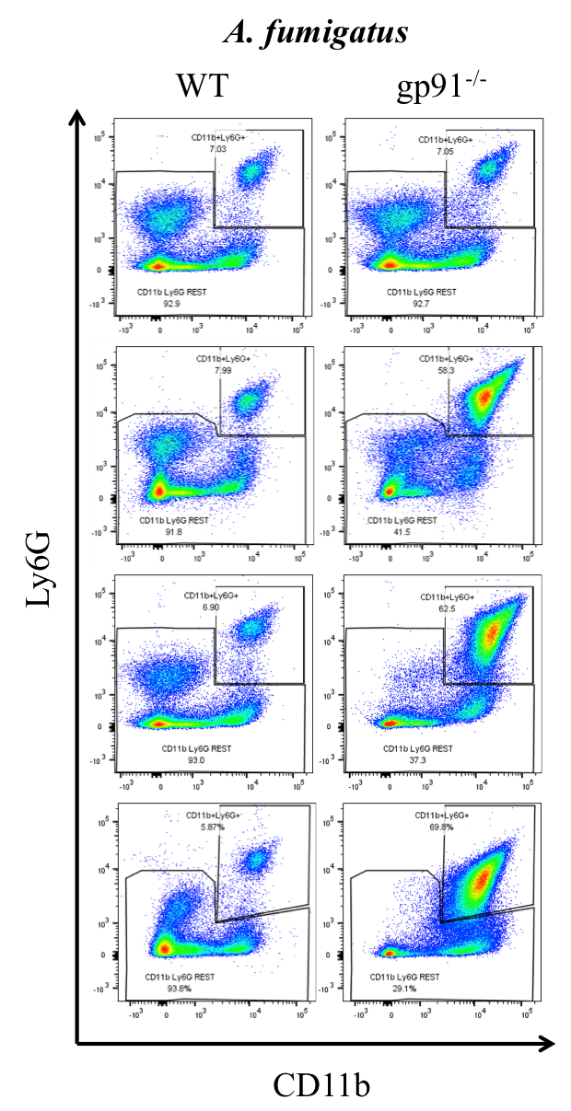

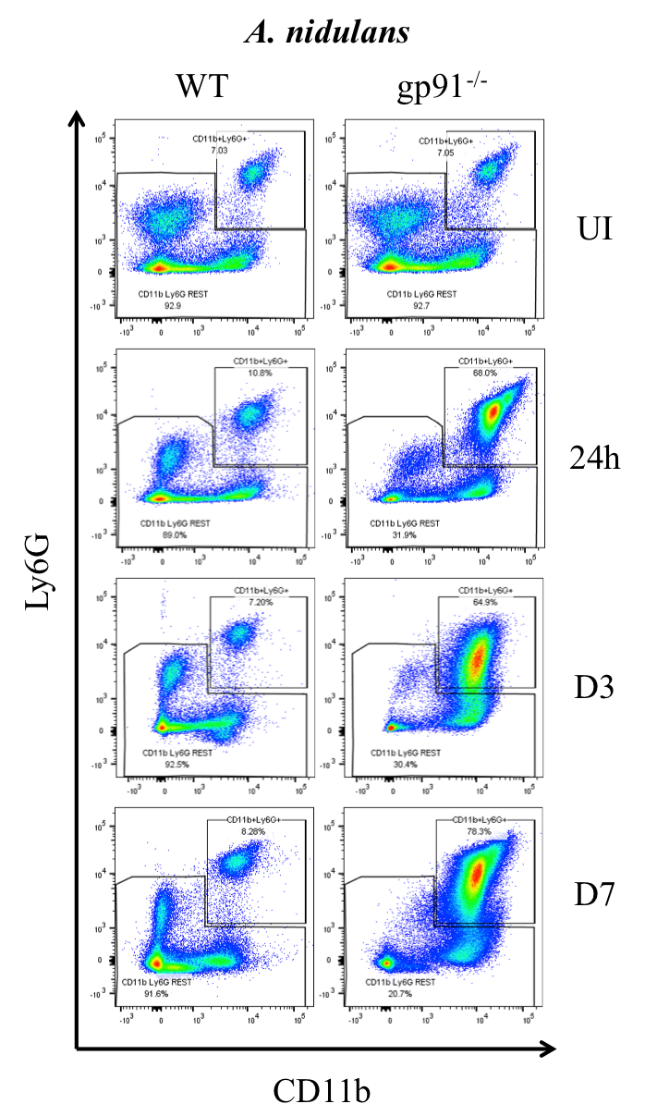


**Suppl. Figure 3. Reduction in alveolar macrophages following *Aspergillus* infection.** Representative FACS plots from WT and gp91­^-/-^ mice at each time point following *A. fumigatus* (left panel) or *A. nidulans* infection (right panel).


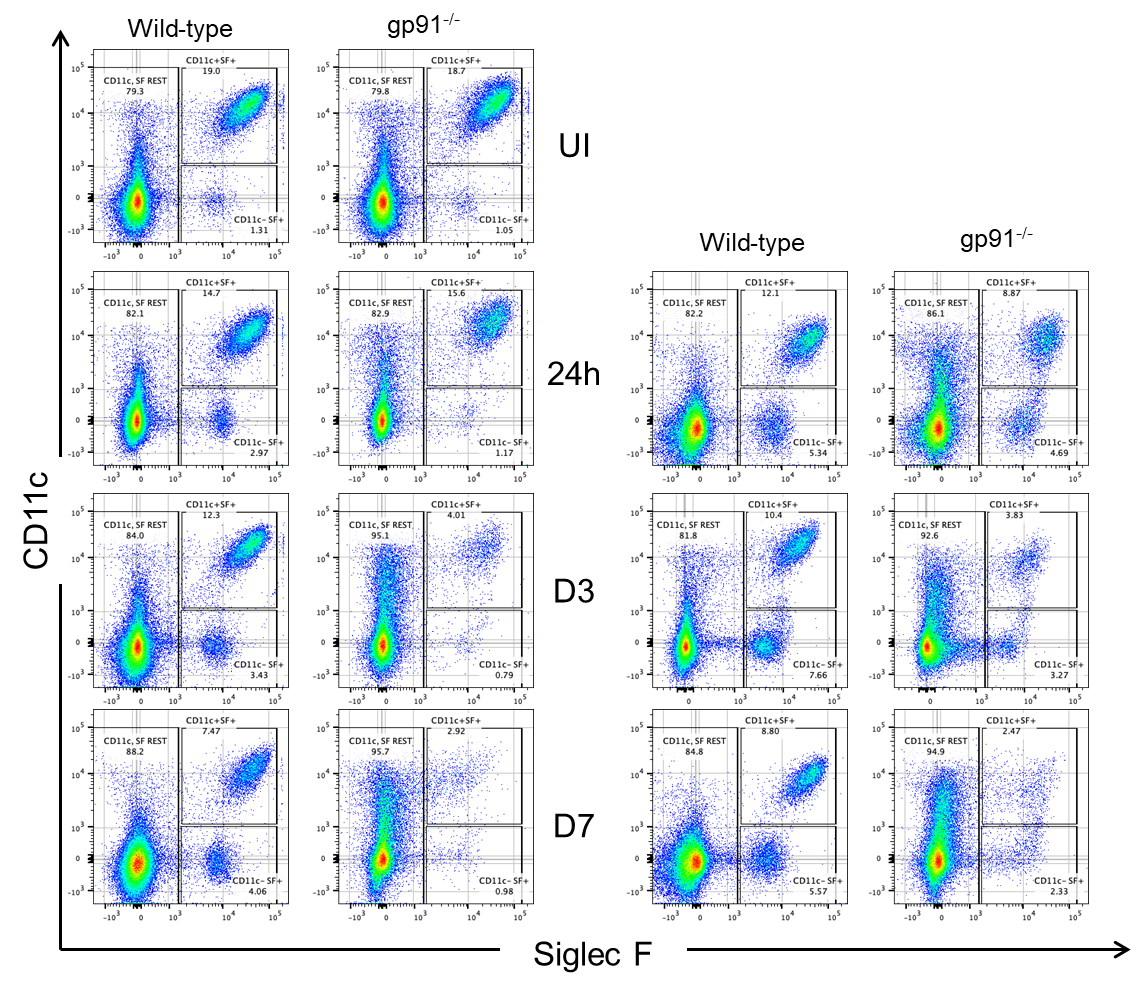


*Aspergillus nidulans*

*Aspergillus fumigatus*

**Suppl. Table 2. Summary of the findings during the characterisation experiments.** 24h, D3, D7 and D17 denote 24 hours, 3 days, 7 days and 17 days post-infection respectively. UI = uninfected; WT = wild-type mice; gp91^-/-^ = CGD mice; cf. = compared with; ↑ = increased, ↓ = decreased; p.i. = pst-infection; = no significant change; *denotes results where data is from a limited number of samples; ns = non-significant.

|  | ***A. fumigatus*** | | ***A. nidulans*** | |
| --- | --- | --- | --- | --- |
|  | **WT** | **gp91^-/-^** | **WT** | **gp91^-/-^** |
| **Mortality** | 0% | 50% at D17 | 0% | 10% at D17 |
| **Weight Loss** | Nil | ≥20% by D2 | Nil | ≥10% by D2 |
| **Histology** | No significant pathology | Extensive multi-focal pulmonary infiltrates by D7  Granuloma formation by D17  Hyphae visible within granulomas. | No significant pathology | Extensive multi-focal pulmonary infiltrates by D7  Granuloma formation by D17 but less well circumscribed than AF  Hyphae visible within granulomas |
| **Pulmonary Immune Cell Recruitment** | | | | |
| **Neutrophils** | = | Marked ↑ by 24h p.i.  Persistently ↑↑ 7d p.i.  Marked ↑ CD11b expression | = | Marked ↑ by 24h p.i.  Persistently ↑↑ 7d p.i.  Marked ↑ CD11b expression |
| **Alveolar Macrophages** | Slight ↓ at D3 & D7  CD11b = | Marked ↓ at 24h, D3 & D7  Increasing CD11b expression following infection | Decreased at 24h, D3 & D7  CD11b = | Marked ↓ at 24h, D3 & D7  Increasing CD11b expression following infection |
| **Interstitial Macrophages** | = | ↑ 24h p.i.  Persistent ↑ at D7 | = | = |
| **Ly6C^hi^ Monocytes/Mφ** | = | ↓ at 24h then returned to baseline | = | ↓ at 24h, ↑ at D3, returned to baseline D7 |
| **Ly6C^lo^ Monocytes/Mφ** | = | ↓ at 24h & D3 before returning to baseline | = | Slight (ns) ↓ at 24h, ↑ at D3, returned to baseline D7 |
| **CD11b^+^ DCs** | = | ↓↓ within 24h  No recovery by D7 | = | ↓ at 24h, D3 & D7, but less marked than AF |
| **CD103^+^ DCs** | = | ↓ D3 & D7 | = | ↓ at D3 & D7 |
| **NK cells** | = | ↓ at 24h, D3 & D7 | = | Marked ↓ at 24h, D3 & D7 |
| **Eosinophils** | Significant ↑ at 24h, D3 & D7  Peak ↑ at D7 | = | Significant ↑ at 24h, D3 & D7  Peak ↑ at D3 | Slight ↑ at D3, returned to baseline by D7 |
| **T cells** | ↑ at D3 | ↓ at 24h, D3 & D7  Nadir at D7 | = | Markedly ↓ at 24h, D3 & D7  Nadir at D3 |
| **Pulmonary Cytokine Response** | | | | |
| **IL-1α** | = | Peaks at D7  (112-fold ↑)  Remains elevated D17 | = | Peaks at D7  (150-fold ↑)  Remains elevated D17 |
| **IL-1β** | = | Steadily ↑ p.i.  166-fold ↑ by D17 | = | Steadily ↑ p.i.  160-fold ↑ by D17 |
| **IL-1α** | = | Peaks at D7  (112-fold ↑)  Remains elevated D17 | = | Peaks at D7  (150-fold ↑)  Remains elevated D17 |
| **IL-1β** | = | Steadily ↑ p.i.  166-fold ↑ by D17 | = | Steadily ↑ p.i.  160-fold ↑ by D17 |
| **IL-6** | = | Peaks at 24h then gradually returns to baseline by D17  76-fold ↑ cf. UI at 24h | = | Biphasic peak at 24h and D7  70-fold ↑ cf. UI at 24h  Returns baseline D17 |
| **CXCL1 (KC)** | = | Peaks at 24h, slowly decreases but remains elevated at D17  40-fold ↑ cf. UI at 24h | = | Peaks at 24h, decreases rapidly by D3  49-fold ↑ cf. UI at 24h |
| **CXCL2 (MIP-2α)*** | = | ↑ at 24h, D3, D7 & D17 | = | ↑at 24h, D3, D7 & D17 |
| **G-CSF*** | = | Peaks at 24h then resolves | = | Maximal at 24h with 2^nd^ peak at D3 |
| **GM-CSF*** | = | Peaks at 24h then resolves | = | Maximal at 24h with 2^nd^ peak at D3 |
| **IL-12** | = | Minimal ↑  Peaks at D7 with a 2-fold ↑ cf. UI | Peaks at D3 with a 12-fold ↑ | Minimal ↑  Peaks at D7 with a 2-fold (ns) ↑ |
| **TNF-α** | Peaks at D17 with a 2-fold ↑ cf. UI | Peaks at D17 with a 2-fold ↑ cf. UI | Peaks at D3 with a 7-fold ↑ cf. UI | Peaks at D7 with a 5-fold ↑ cf. UI |
| **IFN-γ** | = | = | = | Peaks at D7 with a 6-fold ↑ cf. UI |
| **IL-10** | = | Gradual ↑  Maximal at D17 | Gradual (ns) ↑,  Maximal D3 | Gradual (ns) ↑  Maximal at D7 |
| **CCL2**  **(MCP-1)*** | = | Increased by 24h  Peaks at D7 | = | Increased by 24h  Peaks at D7 |
| **CCL3**  **(MIP-1α)*** | = | Increased from 24h  Peaks at D3  (48-fold ↑ cf. UI) | = | Increased from 24h  Peaks at D7  (68-fold ↑ cf. UI) |
| **CCL4**  **(MIP-1β)*** | = | Increased from 24h  Peaks at D7 | Slight (ns) ↑ at D3 | Increased from 24h  Peaks at D3 |
| **IL-33*** | = | Peaks at D17  (14-fold ↑ cf. UI) | = | Peaks at D7  (8-fold ↑ cf. UI) |
| **IL-17** | = | Peaks at D7  (96-fold ↑ cf. UI) | = | Peaks at D7  (77-fold ↑ cf. UI) |
| **IL-23*** | = | Peaks at D17 | Peaks at 24h | Peaks at 24h |
